# RNAi-Based Functional Genomics Identifies New Virulence Determinants in Mucormycosis

**DOI:** 10.1371/journal.ppat.1006150

**Published:** 2017-01-20

**Authors:** Trung Anh Trieu, María Isabel Navarro-Mendoza, Carlos Pérez-Arques, Marta Sanchis, Javier Capilla, Patricia Navarro-Rodriguez, Loida Lopez-Fernandez, Santiago Torres-Martínez, Victoriano Garre, Rosa María Ruiz-Vázquez, Francisco E. Nicolás

**Affiliations:** 1 Departmento de Genética y Microbiología, Facultad de Biología, Universidad de Murcia, Spain; 2 Unidad de Microbiología, Universitat Rovira i Virgili, IISPV, Tarragona, Spain; Geisel School of Medicine at Dartmouth, UNITED STATES

## Abstract

Mucorales are an emerging group of human pathogens that are responsible for the lethal disease mucormycosis. Unfortunately, functional studies on the genetic factors behind the virulence of these organisms are hampered by their limited genetic tractability, since they are reluctant to classical genetic tools like transposable elements or gene mapping. Here, we describe an RNAi-based functional genomic platform that allows the identification of new virulence factors through a forward genetic approach firstly described in Mucorales. This platform contains a whole-genome collection of *Mucor circinelloides* silenced transformants that presented a broad assortment of phenotypes related to the main physiological processes in fungi, including virulence, hyphae morphology, mycelial and yeast growth, carotenogenesis and asexual sporulation. Selection of transformants with reduced virulence allowed the identification of *mcplD*, which encodes a Phospholipase D, and *mcmyo5*, encoding a probably essential cargo transporter of the Myosin V family, as required for a fully virulent phenotype of *M*. *circinelloides*. Knock-out mutants for those genes showed reduced virulence in both *Galleria mellonella* and *Mus musculus* models, probably due to a delayed germination and polarized growth within macrophages. This study provides a robust approach to study virulence in Mucorales and as a proof of concept identified new virulence determinants in *M*. *circinelloides* that could represent promising targets for future antifungal therapies.

## Author Summary

Mucormycosis is an infectious disease caused by organisms of the order Mucorales. It is a lethal infection that is raising the alarm in the medical and scientific community due to its high mortality rates, unusual antifungal drug resistance and its emerging character. Among the reasons explaining the nescience about this disease is the lack of knowledge on the biology of the organisms that cause mucormycosis, which is encouraged by the reluctance of these species to genetic studies. In this work, we have developed an RNAi-based functional genomic platform to study virulence in Mucorales. It is a powerful tool available for the scientific community that will contribute to solve the reluctance of Mucorales to genetic studies and will help to understand the genetic basis of virulence in these organisms. Secondly, and as a proof of concept, we have used this genetic tool to identify two new virulence determinants in *Mucor circinelloides*. Lack of function of these determinants delays germination and growth of spores, conceding time to macrophages for the inactivation of the pathogen. The two genes identified, *mcplD* and *mcmyo5*, represent promising targets for future development of new antifungal therapies against mucormycosis.

## Introduction

Mucormycosis is a fungal infection caused by species of the order Mucorales that represents the third most common angio-invasive fungal infection after candidiasis and aspergillosis. Due to the unusual antifungal drug resistance of Mucorales, mucormycosis is considered one of the most important medical complications in immunocompromised patients [1]. Among current antifungal drugs, fluconazole, voriconazole, posaconazole and itraconazole are potent agents of choice used in aspergillosis and candidiasis that, unfortunately, present poor activity against mucormycosis [2]. More specifically, amphotericin B, an old-known macrolide antifungal compound with severe adverse effects, and more recently isavuconazole are used against mucormycosis although they only achieve partial activity [3–5]. As a consequence of this lack of efficient antifungal drugs, mortality rates of mucormycosis remain higher than 50% and reach up to 90% in disseminated infections [6, 7]. Another negative aspect of mucormycosis is its emerging condition. Only a few years ago, mucormycosis was considered a rare infection limited to immunocompromised patients suffering diabetes, organ transplant or other diseases associated with immunosuppression [8]. However, the current improvement in the diagnostic techniques has revealed an alarming number of mucormycosis cases in immunocompetent patients that have severe trauma (e.g. burn patients, traumatic injuries), since it is now rarely misdiagnosed as aspergillosis [9]. Thus, the isolation of new strains that are capable of infecting healthy individuals and the increasing number of reported cases have raised the alarm on this emerging disease. Together, the lack of effective treatments and the emerging character of this devastating disease are urgently demanding new strategies to prevent and/or treat mucormycosis.

The development of therapies to treat mucormycosis is restricted by the lack of knowledge about the disease and the organisms that cause the infection. One of the main reason explaining the scarce information about mucormycosis is the high reluctance of Mucorales to modern molecular genetics techniques. Among Mucorales, *Rhizopus oryzae* and *Mucor circinelloides* are two study models in which genetic transformation is available [10, 11]. Study of pathogenesis in these two models has revealed iron uptake, spore size, spore coat proteins and dimorphism as virulence determinants in mucormycosis [12–18]. In *M*. *circinelloides*, along with genetic transformation, the application of molecular tools has allowed the dissection of its RNAi mechanism, which has become a useful tool for functional genetics in this fungus [19–22]. Besides its applications as a genetic tool, the RNAi mechanism of *M*. *circinelloides* has a regulatory role that controls complex physiological processes such as growth, sexual and asexual sporulation and death by autolysis [19, 23–27]. Moreover, the extensive study of the RNAi mechanism in *M*. *circinelloides* led to the discovery of the first link between this endogenous regulatory mechanism and the unusual antifungal drug resistance of Mucorales [28]. This novel mechanism generates spontaneous resistance to the antifungal drug FK506 by epigenetic RNAi-mediated post-transcriptional silencing of the *fkbA* gene encoding the protein FKBP12, which is the natural target of FK506. As a result, the lack of target blocks the action of FK506 and the fungus becomes resistant to this drug, suggesting that similar mechanisms could be behind of the exacerbated resistance to antifungal drugs in Mucorales.

The limited knowledge about Mucorales, mainly due to the phylogenetic distance and genetic differences of these basal fungi with other well-known fungi like Ascomycota and Basidiomycota [29], together with the low efficiency of the current antifungal drugs, makes it urgent the development of novel strategies to study this group of organisms and, more specifically, the finding of new virulence determinants that could become future antifungal drug targets in Mucorales. Consequently, the main purpose of this work has been the establishment of a functional genomic approach based on the RNAi mechanism of *M*. *circinelloides* to select phenotypes relevant for the biology of Mucorales and related to virulence, and subsequently to identify the genes responsible for these phenotypes. RNAi has been used as a powerful reverse genetic tool to develop functional whole-genome studies in many organisms, including worms [30], flies [31] and mammal cells [32]. In these reverse genetic approaches, a defined library is laboriously constructed by designing a silencing vector for each annotated gene of the studied organism. In these studies, the model organism requires an easy transformation method ready to be arrayed in large-scale assays in which each silencing vector/molecule is individually delivered. Unfortunately, this is not the case of *M*. *circinelloides* or any other emerging Mucoral. These inconveniences have led us to design a different approach that uses RNAi as a forward genetic tool in which a library representing *M*. *circinelloides* whole genome was constructed in a vector that silenced the cloned inserts. Transformation with this silencing library generated a collection of silenced transformants ready for phenotype screenings in a similar way as in classic chemical or insertional mutagenesis approaches. This transformant collection and the silencing library represent a new genetic tool in Mucorales for forward genetics and functional analysis at whole genome level. Using this approach we have isolated phenotypes related to virulence leading to the identification of two new virulence determinants in *M*. *circinelloides*, the enzyme Phospholipase D (PLD) and a Myosin 5 (Myo5) motor protein, which are required for full virulence in *Galleria mellonella* and *Mus musculus* host models. Overall, this work illustrates a new approach to study virulence in Mucorales at the whole genome level.

## Results

### A genome wide RNAi-based library provides a representative collection of silenced transformants in *M*. *circinelloides*

**Fig 1:**
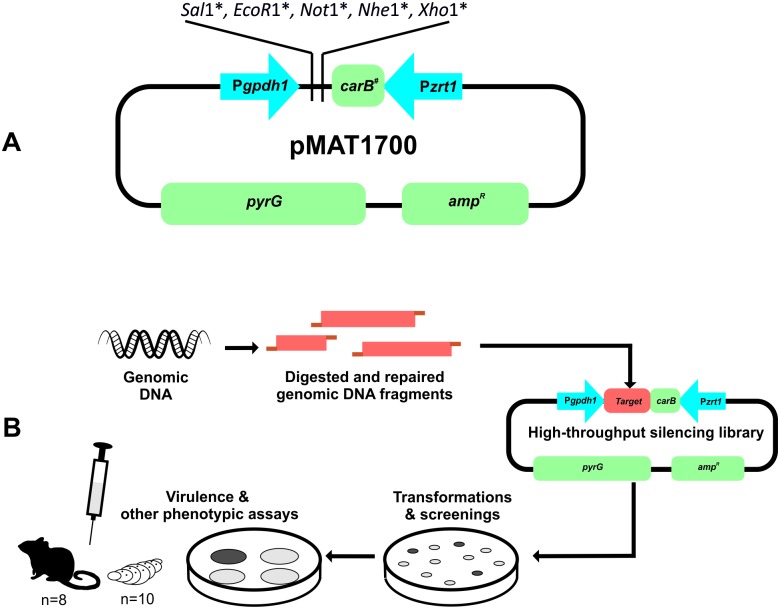
Schematic diagram of the RNA silencing vector pMAT1700 (A) and the platform for functional genomics in *M*. *circinelloides* (B). P*zrt1*, *M*. *circinelloides zrt1* promoter; P*gpd1*, *M*. *circinelloides gpd1* promoter; *carB*^*#*^, 0.5 kb fragment of *M circinelloides carB* gene; *pyrG*, *M*. *circinelloides* orotidine 5'-phosphate decarboxylase gene; *amp*^*R*^, ampicillin-resistant gene. Asterisks indicate unique restriction sites in the vector.

A vector capable of inducing RNAi from any random DNA fragment was designed previously to the construction of the gDNA library for phenotypic screening based on RNAi. RNAi can be triggered in *M*. *circinelloides* by using self-replicative plasmids containing either complete or fragmented genes with their own promoters, obtaining silencing frequencies ranging between 3% and 30% [21]. However, highest silencing frequencies (nearly 95%) can be achieved when the plasmid contains a strong promoter and hairpin structures that directly transcribe dsRNA [22]. To circumvent the limitations of constructing a hairpin producing vector for each gene of *M*. *circinelloides* genome, we designed a high-throughput silencing vector (pMAT1700) with two convergent promoters and no terminator sequences that are flanking a multiple cloning site (MCS) in which random gDNA fragments can be cloned (Fig 1A). In addition, a sequence of 0.5 kb of *carB* gene was cloned next to the MCS to be used as a reporter of silencing (Fig 1A). This gene encodes a phytoene dehydrogenase involved in the production of β-carotene, a pigment responsible for the typical yellow color of *M*. *circinelloides* [33]. Triggering of RNAi after transformation by plasmids containing this reporter produces albino transformants that are easily detectable and also signalize silencing of any other sequence cloned next to it. Fragments of 0.5–4 kb were isolated from *M*. *circinelloides* gDNA partially digested with *Sau3A* and filled in with dGTP-dATP to avoid self-ligation. The genomic fragments thus obtained were ligated with vector pMAT1700 digested with *XhoI* and filled in with dCTP-dTTP to make their ends compatible with *Sau3*A filled fragments and avoid self-ligation, thus favoring the frequency of recombinant clones in the library [34]. Ligation mixtures were introduced into *Escherichia coli* cells to generate the genome-wide RNAi library (Fig 1B) consisting in roughly 83,000 clones, which determined a confidence level higher than 99%. Pooled plasmids were directly purified from the *E*. *coli* colonies and used to transform *M*. *circinelloides* MU402 (*pyrG*^-^ and *leuA*^-^) strain. The empty vector pMAT1700 and a version of this vector lacking the *carB* fragment (pMAT1701) were used as controls to monitor silencing efficiency. Up to sixty transformations following this approach were required to obtain a collection of 51,657 silenced transformants (S1 Table), which ensured a 95% confidence level. Comparison of silencing frequencies obtained with the empty vector and the high-throughput silencing library showed a pronounced increase of *carB* silenced transformants among those obtained with the library (87%) relative to the empty plasmid pMAT1700 (43%) (S1 Table). As expected, the plasmid pMAT1701 did not trigger silencing in any of the transformants (S1 Table). The increase in silencing frequency among transformants obtained with the library could be explained if the 0.5 kb fragment of *carB* gene that is cloned between the two promoters was not long enough in the empty plasmid to allow efficient convergent transcription from both promoters. Nevertheless, once the convergent cassette assimilates new fragments in the library, the silencing efficiency increases close to the maximum previously observed with hairpin triggering molecules [22]. These results demonstrated a high silencing efficiency of our high-throughput library in *M*. *circinelloides*.

### Selection of avirulent phenotypes

**Fig 2:**
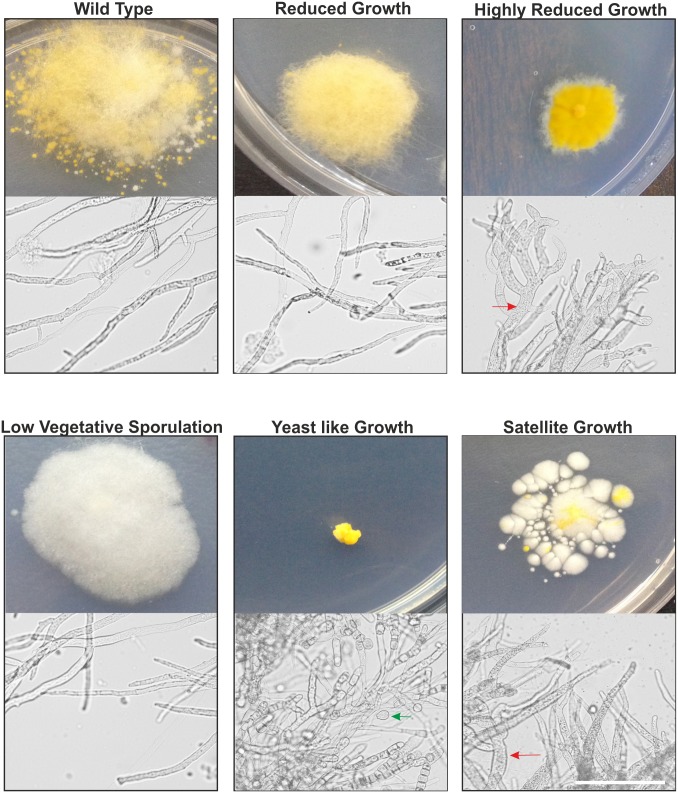
Representative images of wild type strain and the five groups of abnormal phenotypes selected from screenings. Colonial mycelia were grown in MMC medium at pH 3.2 during 72 hours. For the micrographs below each colony, a piece of mycelium from the edge of each colony was observed under the light microscope. Red arrows point to swollen hyphae and green arrow points to yeast cells. Scale bars represent 50 μm.

The main purpose of generating a high-throughput functional genomic tool in *M*. *circinelloides* was to use it as a new approach to find unknown virulence determinants in Mucorales (Fig 1B). In order to find candidate genes involved in *M*. *circinelloides* pathogenesis, we focused the screening of silenced strains on abnormal growth and morphology, since those aspects of fungal physiology have been related to pathogenesis in *M*. *circinelloides* and other fungi [14, 35]. Special attention was paid to transformants growing as yeast-like colonies and showing altered dimorphism and strong reduction of the growth rate, as it is one of the few processes previously associated with virulence in *M*. *circinelloides* [13]. Plates from the transformations with the silencing library were directly screened for abnormal phenotypes (51,657 silenced transformants), resulting in the selection of fifteen transformants with different abnormalities. Later, the transformation plates were further incubated and vegetative spores were pooled together to obtain the collection of silenced spores harboring the high-throughput silencing library described in the previous section. A total of 1x10^4^ viable spores from this collection were grown in new plates for a second screening, resulting in the selection of eleven abnormal candidates. In addition, the second screening confirmed that silencing is maintained in the collection of silenced spores, since the frequency of silenced colonies (79±4% of albino colonies) was similar to the previously described in the original transformants (S1 Table). Growth rate and sporulation efficiency of the twenty six isolated candidates from the two screenings were quantified and classified into five categories based on the different morphological abnormalities that they presented (Fig 2 and Table 1). The first category, the most abundant with 16 isolates, presented a reduced growth (RG1-16) compared with control transformants, but wild-type sporulation. Two transformants presenting a highly reduced growth (HRG1 and HRG2) were included in the second category, as they showed clear differences with the first category, including a reduced vegetative sporulation. The third category comprised five transformants that showed a strong lack of vegetative sporulation (LVS1-5). In addition to the lack of sporulation, some of these five transformants also presented a reduced growth similar to the first category (Table 1). The fourth category contained only one transformant that showed a yeast-like growth (YLG1). This transformant presented the slowest growth, forming small colonies similar to yeasts rather than mycelial colonies. The morphology of YLG1 under the optical microscope also showed strongly deformed cell walls incapable of forming regular filaments (Fig 2). These filaments appeared to be septated, although one of the main characteristics of Mucorales is their coenocytic mycelium. This contradictory observation could be explained if this transformant is immersed in a hyphae-yeast transition state in which the tip of the hyphae produces yeast cells that resemble a septated structure before the yeast cells are liberated (Fig 2, yeast-like growth). The last category included two transformants showing a satellite growth phenotype (SG1 and SG2). These two transformants grew slower than control transformants, producing long sporangia that bent to the media to form new colonies, acquiring this unusual satellite phenotype in transformation plates at pH 3.2 (Fig 2). When SG1 and SG2 were grown in MMC medium at pH 4.5 (a rich medium but selective for uracil auxotrophy, [36]), they showed reduced growth and sporulation, but not the satellite phenotype. HRG and SG transformants also presented abnormal mycelia under the optical microscope, showing swollen hyphae with abnormal branching (Fig 2). Accordingly with the mechanism of silencing previously described in *M*. *circinelloides* [21], the twenty six transformants showed a reversible phenotype and they lost the abnormalities when they were grown in a non-selective medium for several vegetative cycles, confirming that the phenotypes were caused by the silencing of some genes harbored in the plasmids.

**Table 1:** Phenotypic characterization of silenced transformants with abnormal phenotypes.

Strain	Growth rate*	Sporulation^#^	Pathogenicity
WILD TYPE			
MU402+pMAT1700	5.98 ±0.12	5.86±0.40	VIRULENT
REDUCED GROWTH (RG)			
RG1	**4.09±0.13**	5.53±0.15	VIRULENT
RG2	**4.45±0.27**	5.61±0.17	VIRULENT
RG3	**4.26±0.22**	5.62±0.21	VIRULENT
RG4	**5.01±0.19**	5.53±0.31	VIRULENT
RG5	**4.27±0.20**	5.63±0.26	VIRULENT
RG6	**4.05±0.14**	5.43±0.35	VIRULENT
RG7	**4.94±0.22**	5.56±0.11	VIRULENT
RG8	**5.27±0.10**	5.46±0.31	VIRULENT
RG9	**4.15±0.22**	5.41±0.26	VIRULENT
RG10	**3.94±0.17**	5.63±0.25	VIRULENT
RG11	**4.03±0.14**	5.71±0.21	VIRULENT
RG12	**5.01±0.20**	5.75±0.17	VIRULENT
RG13	**4.38±0.23**	5.55±0.45	VIRULENT
RG14	**4.25±0.13**	5.26±0.37	VIRULENT
RG15	**5.01±0.19**	5.43±0.41	VIRULENT
RG16	**4.07±0.33**	5.22±0.26	VIRULENT
HIGHLY REDUCED GROWTH (HRG)			
HRG1	**2.03±0.33**	**4.06±0.20**	**AVIRULENT**
HRG2	**2.04±0.27**	**4.01±0.15**	**AVIRULENT**
REDUCED SPORULATION			
RS1	6.04±0.31	**3.13±0.35**	VIRULENT
RS2	**4.74±0.19**	**4.21±0.31**	VIRULENT
RS3	6.00±0.17	**3.21±0.41**	VIRULENT
RS4	**4.23±0.25**	**4.21±0.26**	VIRULENT
RS5	**7.11±0.26**	**3.46±0.35**	VIRULENT
YEAST LIKE GROWTH (YLG)			
YLG1	**0.48±0.06**	**0.04±0.01**	**AVIRULENT**
SATELLITE GROWTH (SG)			
SG1	**3.19±0.23**	**2.51±0.41**	VIRULENT
SG2	**3.19±0.26**	**2.53±0.25**	VIRULENT

Data are represented as mean ± SEM.

* Diameter (cm).

^#^ Spores x10^6^/(cm^2^). Bold numbers represent significant differences (P<0.01, t-test).

**Fig 3:**
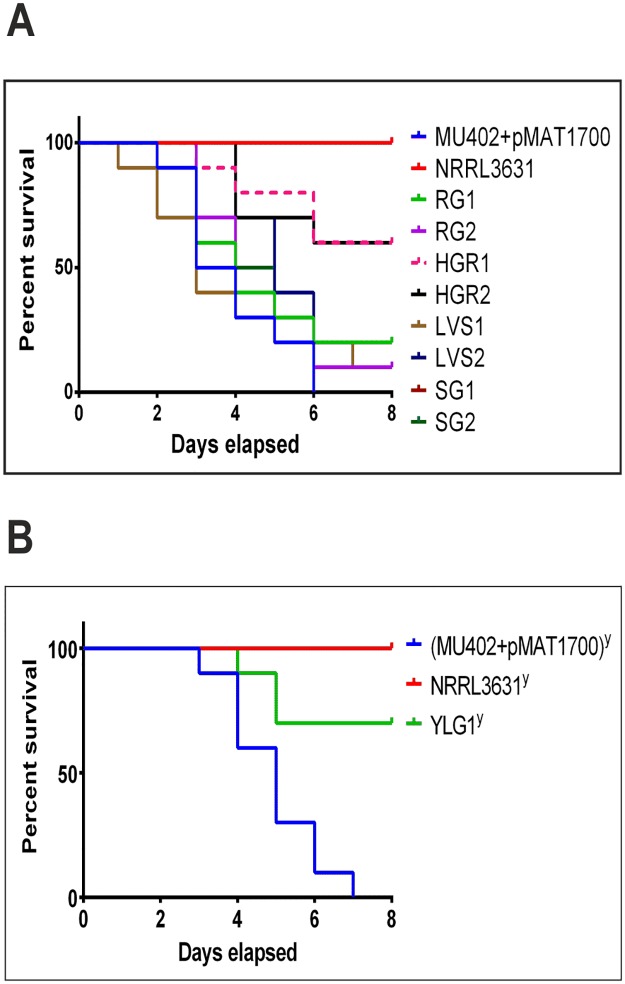
Virulence assays of selected transformants with abnormal phenotypes. (A) Virulence assays of all highly reduced growth (HRG) and satellite growth (SG) transformants, and two representative transformants from reduced growth (RG) and low vegetative sporulation (LVS) phenotypes. Ten *G*. *mellonella* larvae were injected with 2,000 spores of each tested strain. Only transformants HRG1 and HRG2 showed a significant reduced virulence (p = 0.0014 and p = 0.0016, respectively) (B) Virulence assays of wild type strains and the yeast-like growth (YLG) transformant using an inoculum of 20,000 yeast cells in the infections (^y^). The transformants YLG1 showed a significant reduced virulence (p = 0.0017). NRRL3631 and MU402+pMAT1700 were used as avirulent and virulent control strains, respectively. These data are representative of three independent experiments.

As this work focuses on the identification of new virulence determinants in *M*. *circinelloides*, we performed virulence tests with all the selected transformants in a heterologous host, *Galleria mellonella*, which was previously established as a host model for *M*. *circinelloides* [14]. The viability of larvae infected with two thousand spores was monitored at one day intervals for all the transformants except YLG1, which was unable to produce spores for this assay and, therefore, yeast-like cells were used for the infection assay [13] (Fig 3 and S1 Fig). Among the twenty six transformants, only three isolates were significantly less virulent than the virulent control strains, the two HRG transformants (Fig 3A, HRG1 and HRG2) and the single YLG transformant (Fig 3B, YLG1). The isolation of these three transformants with reduced virulence confirmed that our RNAi-based functional genomics strategy can be used to select phenotypes related to virulence and pathogenesis in *M*. *circinelloides*.

### Candidate gene identification and validation of the silencing phenotypes

The self-replicative nature of *M*. *circinelloides* plasmids used to construct the RNAi libraries facilitates the identification of the silencing sequences responsible for the phenotypes in the selected transformants, since library plasmids are maintained as episomes. Thus, the gDNA sequence present in the silencing plasmids can be identified by PCR amplification and sequenced using oligonucleotides flanking the cloning site. Alternatively, silencing plasmid can be re-cloned in *E*. *coli* and sequenced. In order to validate this hypothetical forward genetic approach in Mucorales, five independent transformants (HGR1, HGR2, YLG1, SG1 and SG2) were selected for gene identification and validation of the silencing phenotype. Three transformants (HGR1, HGR2 and YLG1) were selected due to their avirulent phenotype, whereas the transformants presenting the satellite growing phenotype (SG1 and SG2) were selected to demonstrate that genes involved in other physiological processes can also be identified following this approach. Amplifications from gDNA of these five transformants generated PCR products only in YLG1, SG1 and SG2. After purification and sequencing of these PCR products, the DNA sequences were analyzed and compared to the genome database of *M*. *circinelloides* v1.0 and v2.0 (http://genome.jgi-psf.org/Mucci1/Mucci1.home.html and http://genome.jgi-psf.org/Mucci2/Mucci2.home.html, respectively). The two strains sharing the satellite growth phenotype, SG1 and SG2, exhibited both equal size PCR products and DNA sequences, indicating that these two transformants harbored the same plasmid. The analysis of the sequence amplified from this plasmid revealed the presence of three different ORFs: ID 84675 (CLIP-associated proteins (CLASPs), v1.0), ID 156742 (intracellular protein transport, v2.0) and ID 145873 (DNA repair protein RAD51/RHP55, v2.0) (Table 2). The analysis of the sequence obtained from transformant YLG1 also unveiled a DNA insert containing two different ORFs: ID 51513 (myosin class V heavy chain, v1.0) and ID 166338 (no description in either v1.0 or v2.0). For the analysis of transformants HGR1 and HGR2, plasmid re-cloning in *E*. *coli* was required, revealing that both transformants shared a plasmid with the same insert sequence (pMAT1726). In this case, the sequence of the plasmid insert harbored only one ORF, ID 134906, which encoded a Phospholipase D like protein (v2.0). The analysis of the sequences found in the plasmids of the five selected transformants has been summarized in Table 2, which shows that six different candidate genes could be responsible for three selected phenotypes. In order to identify which genes are behind the phenotypes, a silencing validation experiment was performed for each of the six candidate genes. Five new silencing validation plasmids were engineered by cloning a 1 kb fragment of each candidate gene in the MCS of pMAT1700 (Table 2). After transformation of the recipient wild type strain with these five plasmids and pMAT1726, only three plasmids reproduced the three phenotypes previously observed in the original transformants obtained with the high-throughput silencing libraries (Table 2). Silencing of gene ID 84678 resulted in the satellite growing phenotype previously observed in the transformants SG1 and SG2, whereas the yeast like growth phenotype of YLG1 was reproduced only by silencing of gene ID 51513. As expected, silencing of the only gene (ID 134906) found in the transformants HRG1 and HRG2 resulted in the highly reduced growth phenotype.

**Table 2:** Gene identification and phenotypic validations.

Strains	Candidate Genes	Description	Validation Plasmids	Phenotype
HIGHLY REDUCED GROWTH (HRG)				
HRG1 & HRG2	ID 134906^2^	Phospholipase D	pMAT1726	Highly reduced growth
YEAST LIKE GROWTH (YLG)				
YLG1	ID 51513^1^	Myosin class V heavy chain	pMAT828	Yeast like growth
	ID 166338^2^	(Unknown)	pMAT798	Wild type
SATELLITE GROWTH (SG)				
SG1 & SG2	ID 84675^1^	CLIP-associated proteins (CLASPs)	pMAT823	Satellite growth
	ID 156742^2^	Intracellular protein transport	pMAT824	Wild type
	ID 145873^2^	DNA repair protein RAD51/RHP55	pMAT825	Wild type

^(1)^ Version 1.0 of *M*. *circinelloides* genome (http://genome.jgi-psf.org/Mucci1/Mucci1.home.html).

^(2)^ Version 2.0 of *M*. *circinelloides* genome (http://genome.jgi-psf.org/Mucci2/Mucci2.home.html)

**Fig 4:**
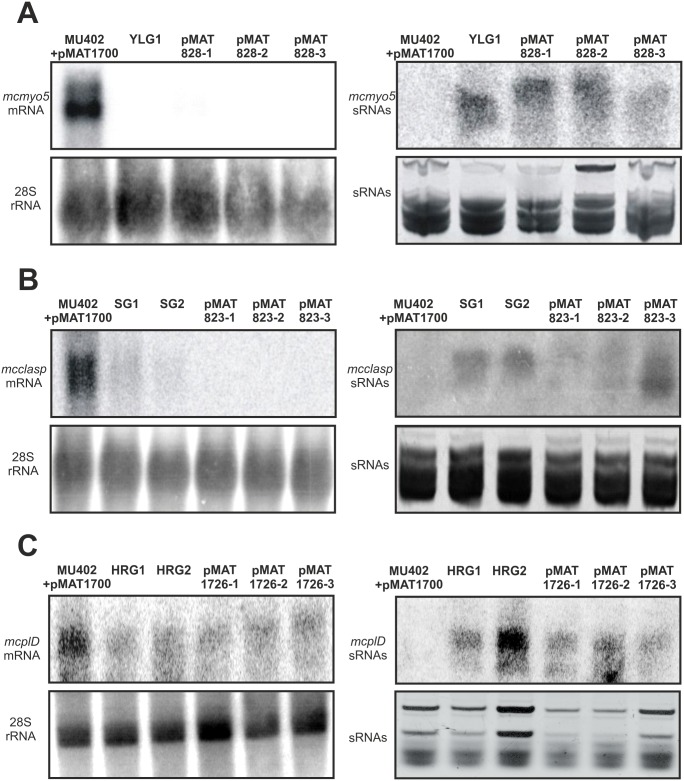
Validation of RNAi-mediated silencing in transformants with selected phenotypes. (A) Levels of *mcmyo5* mRNA and siRNAs in YLG1 original transformant and three transformants carrying a plasmid designed to specifically silence *mcmyo5* (pMAT828). (B) Levels of *mcclasp* mRNA and siRNAs in SG1 and SG2 transformants, and three transformants carrying a plasmid designed to specifically silence *mcclasp* (pMAT823). (C) Levels of *mcplD* mRNA and siRNAs in HRG1 and HRG2 transformants, and three transformants carrying a plasmid designed to specifically silence *mcplD* (pMAT1726). The mRNA loading controls were performed by re-probing the membranes with a 28S rRNA probe, whereas siRNAs loading controls were obtained by Ethidium Bromide staining [21].

To confirm that the phenotypes obtained after the introduction of plasmids harboring sequences of genes IDs 84675, 51513 and 134906 are due to the lack of function of these genes through a canonical RNAi mechanism, we checked the mRNA levels and the production of siRNAs for the three candidate genes in both the original transformants containing the plasmids from the high-throughput RNAi libraries and the transformants obtained with the validation plasmids (Fig 4). All transformants for the three genes showed a reduction of mRNA levels and a production of siRNA for the corresponding gene, confirming the expected mechanism of action of the RNAi high-throughput library. These results demonstrated that the RNAi high-throughput library can be used as a new means to perform forward genetics and functional genomics in the study of virulence of Mucorales.

### Disruption of genes *mcmyo5* and *mcplD* induces morphological abnormalities and avirulent phenotypes in *M*. *circinelloides*

The approach of RNAi-based functional genomics in *M*. *circinelloides* resulted in the identification of two genes that could be new virulence determinants in Mucorales (Fig 3). The role of these two genes was confirmed through the generation of the corresponding knockout strains and the study of their phenotype and virulence in a heterologous host model. The gene ID 51513 (v2.0) encodes a Myosin class V protein that contains the three characteristic domains of this protein family: a motor domain, the IQ motifs and the cargo-binding globular tail. Thus, the gene encoding *M*. *c**ircinelloides* Myosin 5 was denominated *mcmyo5*. In order of adding more evidence to the identity of *mcmyo5* gene, we performed a detailed phylogenetic analysis that included myosin proteins identified in other fungi (S3 Table). This analysis revealed that gene *mcmyo5* encodes a myosin protein that is perfectly clustered among other fungal myosin 5 proteins (S2A Fig). The second gene, ID 134906 (v2.0), encodes a Phospholipase D like protein (accordingly denominated *mcplD*), that contains the characteristic domains (C2, PX, PH), the active site and other functionally important parts of the enzyme [37]. Similarly to *mcmyo5*, we performed a detailed phylogenetic analysis that included phospholipase proteins identified in other fungi (S4 Table). This analysis revealed that gene *mcplD* encodes a phospholipase protein that is perfectly clustered among other fungal phospholipases type D (S2B Fig). In addition, the gene ID 84675 (v1.0) was also mutated, as mentioned above, to prove that the strategy presented in this work is also valid to study other fungal processes different than virulence, and also as a control to prove that not all growth defects are related to reduced virulence. This gene, named as *mcclasp*, encodes a CLIP-associated protein like (CLASPs), as the CLASP N-terminal domain is the main conserved region, which shares an 87% identity with a hypothetical CLASP protein of *Mucor* *ambiguous*. The disruptions of these three genes were carried out through the construction of knockout vectors designed to replace each candidate gene with *pyrG* gene, which was used as a selective marker (S3 Fig). These knockout vectors contained an engineered cassette with adjacent regions of the target genes flanking the *pyrG* gene (S3A Fig) and were used to transform MU402 strain (*pyrG*^-^, *leuA*^-^). After transformations, candidates presenting the phenotype previously associated with silencing of each gene were isolated and the disruptions analyzed by Southern blot analysis (S3B Fig). The two transformants selected from *mcplD* disruption (MU466 and MU467) and the transformant selected from *mcclasp* disruption (MU464) only showed the DNA fragments corresponding to the correct integration of the disruption fragment at the corresponding loci (S3B Fig), indicating that they were homokaryons for the mutant allele. In order to confirm the identity of the product encoded by the gene *mcplD*, activity of the enzyme PLD was measured in the mutant *ΔmcplD* and compared to the wild type strain (“Phospholipase D Assay Kit”, from Sigma-Aldrich). This assay showed a significant reduction (p = 0.0017) of PLD activity of almost 30% in the mutant strain, but not a total lack of PLD activity (S4 Fig). These results could be explained if there are other proteins with similar activity in the crude extracts of this mutant. Regarding the deletion of *mcmyo5* gene, two transformants showing the yeast-like growth phenotype were selected after transformation with a replacement cassette for the gene *mcmyo5*. One of these transformants, MU468, probably harbored a chromosomal rearrangement at the *mcmyo5* locus, whereas the second one, MU465, showed the correct *pyrG* insertion of 4.1 kb replacing *mcmyo5* gene (S3B Fig). However, it was impossible to obtain a homokaryotic knockout strain for the gene *mcmyo5*, as the transformant containing the mutant allele maintained some wild type nuclei even after ten vegetative cycles on selective media (3.4 kb fragment in S3B Fig). These results suggested that *mcmyo5* gene may play an essential role in the viability of *M*. *circinelloides* and a homokaryotic state of the mutant nuclei might be lethal. The heterokaryotic strain containing *mcmyo5* mutant nuclei was named *Δmcmyo5*^*(-)(+)*^.

**Fig 5:**
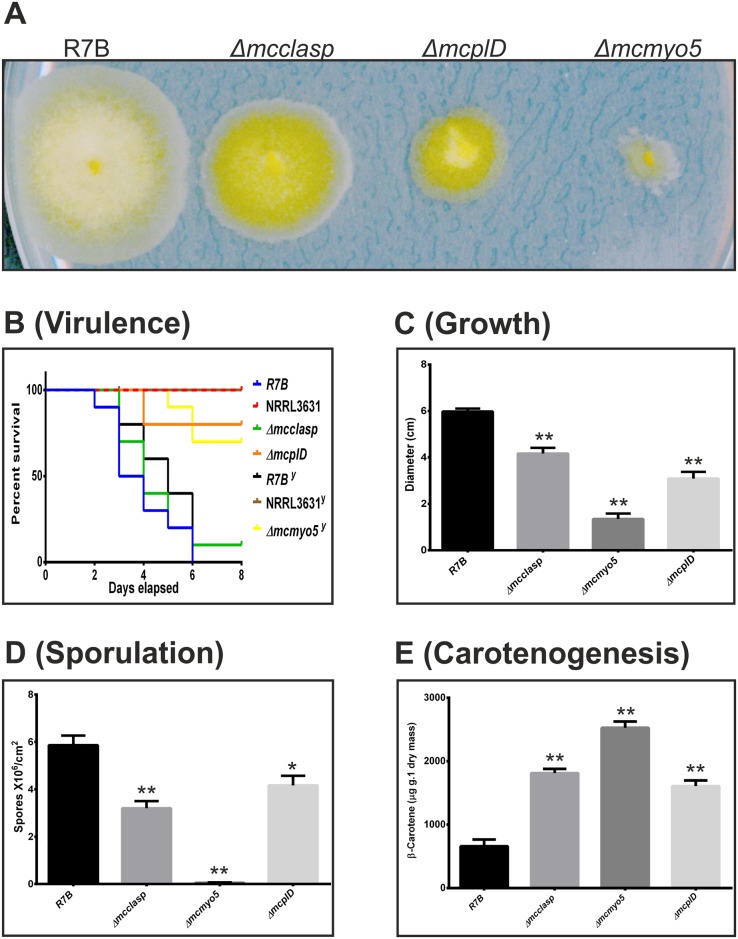
Phenotypic analyses of *mcclasp*, *mcmyo5* and *mcplD* knockout mutants. (A) Colonies of the three mutants and a wild type strain (R7B) cultivated in rich medium MMC at pH 4.5 for 48 h. (B) Virulence assays using spores of *mcclasp* and *mcplD* knockout mutants, and yeast cells from the *mcmyo5* (^y^) heterokaryon mutant in the heterologous host model *G*. *mellonella*. NRRL3631 and R7B (spores and yeast cells) were used as avirulent and virulent control strains, respectively. Quantification of growth rate (C), vegetative sporulation (D) and β-carotene production (E) was performed in the same growth conditions as in (A). Values are means and standard errors of 10 independent experiments.

**Fig 6:**
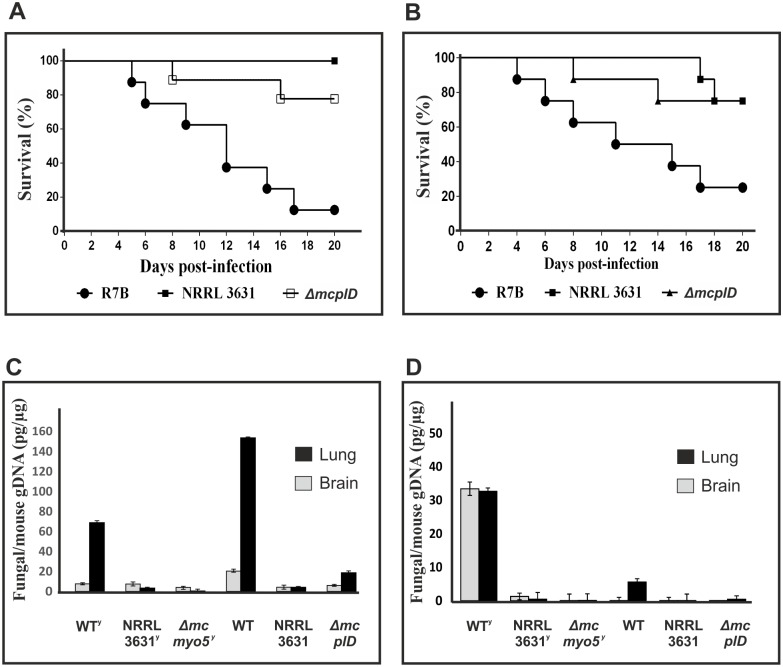
Virulence tests of the *ΔmcplD* and *Δmcmyo5*^*(-)(+)*^ mutants of *M*. *circinelloides* in a murine host model. (A) Virulence assays using spores of wild type strains and the mutant *ΔmcplD*. Mice were injected with 1x10^6^ sporangiospores. (B) Virulence assays using yeast cells of wild type strains and the mutant *Δmcmyo5*^*(-)(+)*^. Injections contained 1x10^6^ yeast cells. (C) Quantification analyses of fungal DNA in mice target organs after two days post infection. (D) Quantification analyses of fungal DNA in mice target organs after five days post infection. Infections were carried out with yeast (y) or spore inocula forms of the different strains of *M*. *circinelloides*.

The phenotype of each knockout strain was equivalent to those observed in the silencing transformants (Fig 5A). The three mutants showed a reduction of growth and sporulation rates, as well as an increase in the production of β-carotene (Fig 5C, 5D and 5E, respectively). The accumulation of β-carotene in the three mutants might be due to the growth stress present in these strains, since diverse stress factors have been previously linked to the production of β-carotene in other organisms [38]. Regarding virulence, infection assays in *G*. *mellonella* larvae with sporangiospores from mutants *ΔmcplD* and *Δmcclasp*, and yeast cells from mutant *Δmcmyo5*^*(-)(+)*^, revealed a significant reduction in virulence of mutants *Δmcmyo5*^*(-)(+)*^ and *ΔmcplD* but not in *Δmcclasp* (p = 0.0007, p = 0.0002 and p = 0.4420, respectively) (Fig 5B), as expected from the results previously obtained with the strains containing silencing vectors (Fig 3). The moth *G*. *mellonella* is a convenient model to study virulence when numerous candidates have to be tested. However, the immune system of this invertebrate model presents several differences compared to vertebrates, especially with warm blooded animals like mammals. Thus, the avirulent phenotype of mutants *Δmcmyo5*^*(-)(+)*^ and *ΔmcplD* was tested in a mouse model, where temperature and immune system components and action mechanisms are similar to humans. Yeast cells and spores of mutants *Δmcmyo5*^*(-)(+)*^ and *ΔmcplD* (respectively) were injected in immunodepressed mice and survival was daily monitored during twenty days after the infection with inocula of both 1x10^5^ (S5 Fig) and 1x10^6^ (Fig 6). Both inocula generated similar results, confirming the avirulent phenotype of mutant *ΔmcplD* in the murine model with a strong statistical significance (p = 0.0081 in S5A Fig and p = 0.0065 in Fig 6A). However, although a reduced virulence was also observed for the heterokaryotic strain *Δmcmyo5*^*(-)(+)*^ compared to the wild type R7B strain, difference was not statistically significant (p = 0.1595 in S5B Fig and p = 0.0526 in Fig 6B). This was probably due to the long time-course of the virulence assay in mice, since heterokaryotic *Δmcmyo5*^*(-)(+)*^ cells might segregate to a wild type phenotype by losing mutant nuclei when grown under non-selective conditions. In fact, growing of mutant *Δmcmyo5*^*(-)(+)*^ in non-selective culture medium gave rise to patches of wild-type phenotypes after few days of incubation (S6A Fig). To test this hypothesis, retrieved CFUs from infected organs of both agonizing mice that showed signs of an imminent death and apparently healthy mice were analyzed in a Southern blot assay that distinguishes between the mutant and wild type genotypes (S6B Fig). Quantification of the proportion of wild type and mutant nuclei in these retrieved CFUs showed correlation between the segregation to wild type genotype and the restitutions of virulence, which supported the role of the gene *mcmyo5* in the pathogenesis of *M*. *circinelloides* (S6C Fig). In order of acquiring more insights about the virulence of the strains *ΔmcplD* and *Δmcmyo5*, the fungal burden was quantified in the relevant organs of mice infected with wild type R7B and both mutant strains. Quantification of fungal gDNA on relevant target organs (brain and lung) revealed prevalence of R7B in tissues from mice infected with both yeast and spore forms, showing a more significant presence in lung tissues (Fig 6C and 6D). In particular, the presence of R7B in lung tissue was higher at day 2 post infection (Fig 6C) than at five days (Fig 6D), indicating a decrease of fungal biomass in mice over time. Such fungal burden decrease was less accentuated on infection with R7B yeasts, suggesting that this fungal form is more persistent in mice during the infection progression. Despite these differences in fungal load, symptoms and mortality rates were similar after infection with R7B spores and yeasts (Fig 6A and 6B). Conversely, a low amount of fungal DNA in mice infected with NRRL3631 and mutant strains *ΔmcplD* and *Δmcmyo5* was detected, even below the limit of detection (0.005 ng) after five days of infection (Fig 6C and 6D). These results, together with survival outcomes, indicated a greater capacity of R7B strain to infect and invade mice tissues, causing higher mortality rates than the mutants *ΔmcplD* and *Δmcmyo*.

### Delayed germination and reduced polar growth as new virulence determinants in mucormycosis

**Fig 7:**
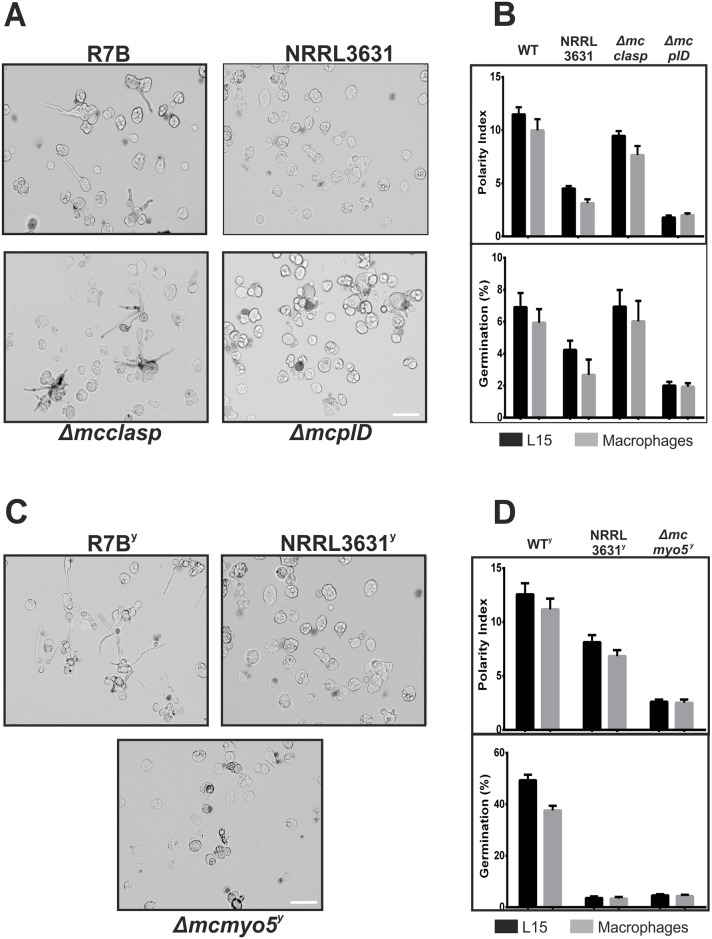
Interaction of macrophage cells and *mcclasp*, *mcmyo5* and *mcplD* knockout mutants. (A) Representative optical microscopic images of the interaction between macrophage cells (J774A.1) and spores of *mcclasp* and *mcplD* mutants. Scale bars represent 50μm. (B) Quantification of germination and polar index of *mcclasp* and *mcplD* mutant spores after four hours of interaction in the presence or absence of macrophage cells. (C) Representative optical microscopic images of the interaction between macrophage cells and yeast cells of the *mcmyo5* knockout mutant. (D) Quantification of germination and polarity index of *mcmyo5* mutant yeast cells after four hours of interaction with macrophage cells. NRRL3631 and R7B were used as avirulent and virulent control strains, respectively. Values are means and standard errors of 500 independent measurements.

The determinants of virulence in *M*. *circinelloides* have been studied during the initial interaction of spores with macrophages, in which the main factor distinguishing virulent and avirulent strains was the size of the spores [14]. Therefore, spore and yeast cell sizes of virulent and avirulent strains were determined. The size of yeast cells produced by the avirulent control strain NRRL3631(+) was pronouncedly reduced compared with yeasts produced by virulent control strain R7B(-) (S7B Fig), in the same manner as occurred with the size of the spores [14] (S7A Fig). However, the sizes of the spores or yeast cells of the mutant strains *ΔmcplD*, *Δmcmyo5*^*(-)(+)*^ and *Δmcclasp* were not significantly reduced when compared to the virulent strain R7B (S7 Fig). These results suggested that the reduction of virulence in the strains *ΔmcplD* and *Δmcmyo5*^*(-)(+)*^ might be due to other factors that are independent of the initial size of the fungal spore or yeast cells. In order to find these factors, the interaction between macrophages and the mutant strains *ΔmcplD* and *Δmcmyo5*^*(-)(+)*^ was also studied. Spores and yeast cells (from *ΔmcplD* and *Δmcmyo5*^*(-)(+)*^, respectively) were co-cultured with the mouse macrophage cell line J774A.1 (ATCC, TIB-67), during four hours. At this time of interaction, all the spore/yeast cells have been phagocytized by macrophages and virulent strains initiate germination and polar growth trying to escape before being inactivated [14]. Thus, we quantified the germination rate and polarity index (a quotient between cell length and cell width [39]) as a measure of virulence of the different strains tested here. A germination delay and a reduced polarity index were observed in mutants *ΔmcplD* and *Δmcmyo5*^*(-)(+)*^ relative to wild type (Fig 7). Mutant *ΔmcplD* presented a germination delay and polarity index similar to the avirulent strain NRRL3631, whereas mutant *Δmcmyo5*^*(-)(+)*^ showed a reduction of the polarity index even more pronounced than the avirulent control strain, as well as a similar germination delay. Mutants grown in absence of macrophages also presented the same delay in germination and polar growth. The knockout strain in the *mcclasp* gene showed a non-significant reduction of the polarity index and no changes in the germination rate when compared to the wild type. As mutant *Δmcclasp* is not affected in virulence, these results highlight the relevance of spore germination and hyphal growth rates within macrophages for *M*. *circinelloides* virulence.

## Discussion

Here, we have developed a new approach based on RNAi high-throughput libraries that allows the identification of genes responsible for virulence in *M*. *circinelloides*. This work represents the first application of a functional genomic approach to identify virulence determinants in Mucorales. RNAi-based reverse genetics has allowed successful whole-genome functional studies in animals (see Introduction), but this technology presents several restrictions in fungi that have prevented its use at whole genome level. Following this reverse genomic approach, the only known study carried out in fungi was firstly reported in a plant pathogen, *Magnaporthe oryzae*, in which the function of a calcium-signaling family of 37 genes was studied using silencing plasmids for each gene of this family [40]. Our platform presents an opposite approach in which a collection of phenotypes are generated by transformation with a whole-genome RNAi library and afterward the genes responsible for a particular phenotype are identified. This approach represents the first forward genetic strategy to study gene function at the whole genome level in Mucorales. Following this strategy, a general screening of a collection of silenced *M*. *circinelloides* transformants led us to the isolation of twenty-six strains showing a wide range of distinct phenotypes in fungal processes such as virulence, growth and sporulation. One advantage of this approach versus traditional chemical or insertional mutagenesis is that essential genes could be isolated, since RNAi usually reduces the expression of the target gene rather than a total inhibition. Another advantage of this RNAi-based functional genomic platform is the possibility of designing conditional screenings. The screenings shown here were performed under non-specific growth conditions, as it was intended to prove the general utility of the platform to apply functional genomics and to show how genes related to virulence can be identified from general screenings. However, the collection of silenced transformants offers the opportunity to carry out specific screenings under particular conditions to select phenotypes related to virulence, such as yeast growth, thermotolerance, protease over-production, etc. Moreover, another attractive advantage of this approach is its exportability to other Mucorales or fungi from different groups like Ascomycota and Basidiomycota, since the only conditions required are the existence of a functional RNAi mechanism and an efficient transformation method, which are both present in many fungal groups [41].

The application of the RNAi-based functional genomic platform has facilitated the identification of the genes *mcplD* as an essential factor to maintain full virulence in *M*. *circinelloides*, both in a heterologous model like *G*. *mellonella* and a murine host model. Knockout mutants in *mcplD* showed deficient growth accompanied with sporulation reduction and increased production of β-carotene, and more importantly, reduced virulence in the host models *G*. *mellonella* and *M*. *musculus*. The gene *mcplD* codes for a Phospholipase D enzyme (PLD), a well-known protein that is highly conserved in different organisms. This enzyme catalyzes the hydrolysis of the phosphodiester bond of glycerophospholipids to generate phosphatidic acid and a free headgroup [37]. Phosphatidic acid functions as an intracellular lipid messenger that activates different target kinases, which in turn activate a broad range of cellular processes such as receptor signaling, control of intracellular membrane transport, and reorganization of the actin cytoskeleton [37]. This pleiotropic function of PLD could explain the complex phenotype observed in the *M*. *circinelloides* mutant for the *mcplD* gene. In addition, PLD has been described as a major virulence factor in *Corynebacterium pseudotuberculosis*, being involved in macrophage death and systemic dissemination of this pathogen [42]. In fungi, *Aspergillus fumigatus* PLD regulates its internalization into lung epithelial cells, and the *pld* gene of *Purpureocillium lilacinum* is significantly up-regulated during infection of *Meloidogyne incognita* eggs [43, 44]. In *M*. *circinelloides*, *mcplD* may regulate some signaling pathways involved in germination and hyphal growth, since mutants in this gene showed delayed germination and reduction of the polarity index. The other gene related to virulence that has been found in this study, *mcmyo5* encodes a processive cargo transporter belonging to the Myosin V Class (Myo5). Myosins play important roles in morphogenesis of filamentous fungi, since they are involved in the establishment and/or maintenance of polarity [45]. Among Myosins, Myo5 supplies a constant transport of organelles, membranous cargo, secretory vesicles, mRNA, lipid and protein vesicles on actin tracks [46]. In *M*. *circinelloides*, Myo5 might play an essential role in viability, since the knockout mutant was viable only as a heterokaryon containing a small proportion of wild type nuclei. This heterokaryotic strain was unable to produce regular hyphae and presented a yeast-like phenotype with no polar growth. Likely, the lack of a continuous transport mediated by Myo5 impairs the correct formation of the mycelium. Since filamentous growth is a major determinant of virulence in *M*. *circinelloides* [13], the yeast-like knockout strain *Δmcmyo5*^*(-)(+)*^ presented a pronounced reduced virulence in *G*. *mellonella*. Similarly, in the dimorphic plant pathogenic fungus *Ustilago maydis*, a single Myosin class V protein encoded by *myo5* was involved in hyphal growth and pathogenicity [47]. However, the reduction of virulence shown by the *Δmcmyo5*^*(-)(+)*^ heterokaryotic mutant in a murine host model did not reach the significance level stablished, although the reduction in the fungal burden was similar to the mutant *mcplD*. These partially contradictory results obtained from the two host models could be precisely due to the heterokaryotic state of mutant *Δmcmyo5*^*(-)(+)*^. The virulence assays in *G*. *mellonella* are performed during eight days, whereas in *M*. *musculus* the assays are prolonged until the twentieth day, which could be time enough for the heterokayotic strain under non selective conditions to segregate and lose mutant nuclei, reverting to the wild type phenotype. According to this hypothesis, wild type patches segregating from *Δmcmyo5*^*(-)(+)*^ mutant cells growing under non selective culture conditions are easily observed after several days of incubation. In addition, a correlation between the segregation to wild type genotype and the restitutions of virulence was observed after genotyping the nuclei proportion of several retrieved CFUs, which further supported a role of gene *mcmyo5* in the virulence of *M*. *circinelloides*. Similar results were obtained in *Rhizopus oryzae* when *FTR1* gene was disrupted by double cross-over homologous recombination, but multinucleated *R*. *oryzae* could not be forced to segregate to a homokaryotic null allele [48]. The heterokaryotic strain *Δmcmyo5*^*(-)(+)*^ showed the strongest reduction of polar growth and germination rates when phagocyted by macrophages, since it was tested during only four hours in non-selective medium, which is not time enough for segregation. Our results from the *in vitro* analysis and the intravenous infection model showed the potential role of *mcmyo5* in virulence of *M*. *circinelloides*. Although Myo5 is a highly conserved protein, which disqualifies it as a specific antifungal target, the fungal cargo domain and the proteins that interact with this domain could represent a promising target for future antifungal developments.

The analysis of germination and polar growth of *Δmcmyo5*^*(-)(+)*^ *and ΔmcplD* mutants and their interaction with mouse macrophages revealed a delayed germination and reduced polarity index of those strains relative to the wild type strain, although the size of the infecting particles (spores or yeast cells) was not reduced in these mutants. These results suggested that a big size of the infecting particle (spore or yeast cells) is not enough to counteract a delayed germination and reduced polarity index. Time of germination and polarity index are two values that measure the velocity of the pathogen growing inside the macrophage and escaping from it. Thus, a possible explanation of the reduced virulence observed in *ΔmcplD* and *Δmcmyo5*^*(-)(+)*^ mutants might be that delayed germination and reduced polarity index concede macrophages time enough to inactivate the pathogen before it escapes from its cytoplasm. Along with the size of the spore previously described [14], our work demonstrated that the time required for germination and the hyphal elongation rate, measured as polarity index, are two new factors to be considered in the analysis of *M*. *circinelloides* virulence.

Besides genes related to virulence, a third gene named *mcclasp* was selected from the RNAi-based functional genomic screenings for further studies. Mutant *Δmcclasp* presented a complex phenotype affecting several fungal processes like vegetative growth, carotene production and sporulation in a similar manner to the *Δmcmyo5*^*(-)(+)*^ *and ΔmcplD* mutants, although the *Δmcclasp* strain was not affected in virulence. Besides that, the main differences between *Δmcclasp* and those mutants were the formation of sporangiophores, which were normal in length in *Δmcclasp* mutants, allowing the formation of satellite colonies in low pH media, along with a less pronounced reduction of growth and sporulation. The gene product of *mcclasp* is similar to CLASP proteins that are involved in the attachment of microtubules to the cell cortex in animals and plants, thereby contributing to self-organization of cortical microtubules [49]. During mitosis, CLASP proteins control the interactions of astral microtubules with the cell cortex, helping the proper positioning and orientation of the spindle [50]. This important role of CLASP proteins during cell division might be behind the reduced growth and decreased sporulation observed in the *Δmcclasp* mutants. In addition, the role of CLASP proteins in the stability of microtubules is essential for the motility of motor proteins, such as kinesins and dyneins. Kinesins participate in the maintenance of the polarity of filamentous fungi (reviewed by Harris, 2006), which could explain the reduction in the polarity index of the *Δmcclasp* mutant relative to the wild type strain. However, unlike Myo5, kinesins does not seem to be involved in polarity establishment in *M*. *circinelloides*, since strains without CLASP protein are still able to generate hyphal growth, although their elongation rate is lower than the wild type strain. Mutant *Δmcclasp* showed a reduced growth rate and vegetative sporulation and an increase in β-carotene production compared to control strain R7B (Fig 5C, 5D and 5E, respectively), similarly to the phenotypes observed in *ΔmcplD* and *Δmcmyo5* mutants. However these phenotypes are not associated with reduced virulence in mutant *Δmcclasp*, indicating that growth defects are not necessarily linked to attenuated virulence and suggesting a possible specific role of *mcplD* and *mcmyo5* genes in pathogenesis. The identification and analysis of *mcclasp* gene demonstrated that along with virulence, other fungal processes can be studied and genetically dissected with the RNAi-based functional genomic platform developed in this work.

In summary, the absence of classic molecular genetic tools and the scarce information about virulence and pathogenesis in Mucorales encouraged us to develop a robust system for RNAi-based functional genomics in *M*. *circinelloides*. It is a new genetic tool that can be used in the study of a wide range of biological processes, including the identification and study of genes related to virulence and pathogenesis. As a result of its implementation, we have identified new virulence determinants in Mucorales that could represent new targets for future antifungal therapies.

## Materials and Methods

### Strains, growth and transformation conditions

The leucine auxotroph R7B, derived from the (-) mating type *M*. *circinelloides* f. *lusitanicus* CBS 277.49 (syn. *Mucor racemosus* ATCC 1216b), was used as the wild type strain. Strain MU402 is a uracil and leucine auxotroph derived from R7B used as recipient strain of the silencing library [36]. The *M*. *circinelloides* f. *lusitanicus* strain of the (+) mating type NRRL3631 was used in virulence assays as an avirulent control.

*M*. *circinelloides* cultures were grown at 26°C in complete YPG medium or in MMC medium as described previously [36]. Media were supplemented with uridine (200 μg/ml) when required. The pH was adjusted to 4.5 and 3.2 for mycelial and colonial growth, respectively. Transformation was carried out as described previously [10]. Macrophage cells, J774A.1 (ATCC, TIB-67), were cultured in L15 medium (Capricorn Scientific GmbH) supplemented with 10% FBS at 37°C and without CO_2_ supplementation.

### Plasmids and genomic libraries

Plasmid pMAT1726 was recovered from gDNA of HRG1 and HRG2 transformants. In order to construct dsRNA-expressing vectors with the target candidate genes, plasmid pMAT1700 was used as cloning vector. Insert fragments corresponding to the 5’ end of each candidate gene (0.5–2 kb) were amplified with primers containing *Not*I and *Xho*I restriction sites to facilitate cloning into pMAT1700 (S2 Table). Plasmid pMAT828 harbors a 2 kb fragment of gene ID 51513 which was PCR-amplified using primer pairs FYL1 and RYL1 (S2 Table). Plasmid pMAT798 contains a 0.9 kb fragment of gene ID 166338 amplified by PCR reactions using primer pairs FYL1.2 and RYL1.2 (S2 Table). To identify the candidate gene responsible for the satellite growth phenotype, three different plasmids were constructed: pMAT823, pMAT824 and pMAT825. These plasmids contain 1.2 kb, 0.9 kb and 0.5 kb fragments corresponding to genes ID 84675, ID 156742 and ID 145873, which were amplified using primer pairs FYL10.1/RYL10.1, FYL10.2/RYL10.2 and FYL10.3/RYL10.3, respectively (S2 Table).

To calculate the coverage of the genomic libraries in the genome of *M*. *circinelloides* and confidence levels, we followed the formula: N = ln(1-P)/ln(1-f); where N is the necessary number of recombinants, P is the desired probability that any fragment of the genome is represented in the library at least one time, and f is the fractional proportion of the genome in a single recombinant. “f” can be further shown to be f = i/g, where i is the insert size and g is the genome size [51].

To disrupt the candidate genes, a *pyrG* selective marker (2 kb fragment amplified from gDNA using primers F-*pyrG* and R-*pyrG* (S2 Table) was fused with adjacent sequences of the candidate coding regions using fusion PCRs, generating a gene replacement fragment. This fragment was cloned into pGEMT-easy vector (Promega) and used to disrupt the candidate genes via homologous recombination. Plasmid pMAT833 was constructed to disrupt the *mcclasp* gene. It contains a 4.2 kb fragment that includes the *pyrG* gene flanked by 1.3 kb of upstream and downstream sequences of *mcclasp* gene, amplified with primers FYL10U/RYL10-*pyrG* and FYL10-*pyrG*/RYL10D (S2 Table), respectively. The 4.2 kb fusion fragment was amplified with internal primers FYL10 and RYL10 (S2 Table). Plasmid pMAT832 was constructed to disrupt *mcmyo5* gene, following the same strategy described for pMAT833, but using primers FYL1U/RYL1-pyrG and FYL1-pyrG/RYL1D to amplify 1.3 kb of upstream and downstream sequences of the *mcmyo5* gene, respectively (S2 Table). In the case of gene *mcplD*, plasmid pMAT1733 was constructed also following the same fusion strategy, but with the specific primers FPLD/RPLD-*pyrG* and RPLD/FPLD-*pyrG*, for two fragments of 0.95 kb from upstream and downstream regions of *mcplD* (S2 Table), respectively.

### Nucleic acid manipulation and analysis

Genomic DNA from *M*. *circinelloides* mycelia was extracted as previously described [36]. Recombinant DNA manipulations were performed as reported [52]. Total RNA was extracted from mycelia grown during 48 hours at 26°C in liquid MMC pH 4.5 medium under light conditions using RNeasy Plant Mini Kit following the supplier’s recommendation (Qiagen). Southern blot and Northern blot hybridizations were carried out under stringent conditions [22]. DNA probes were labeled with [α-32P] dCTP using Ready-To-Go Labeling Beads (GE Healthcare Life Science). For Southern and Northern blot experiments, DNA probes were directly amplified from genomic DNA using the primer pairs FPLD/RPLD-pyrG, FYL10/RYL10-pyrG and FYL1N/RYL1-pyrG for genes *mcplD*, *mcclasp* and *mcmyo5*, respectively (S2 Table). For siRNA analysis, small RNA samples were extracted from mycelia grown 72 hours in liquid MMC medium pH 4.5 at 26°C using the miRVana kit (Ambion), following the instructions of the supplier. Northern blots for siRNAs were performed as previously described using antisense specific riboprobes generated by *in vitro* transcription of the DNA probes described above (MAXIscriptsT7, Ambion) [21]. Quantifications of signal intensities were estimated from autoradiograms using a Shimadzu CS-9000 densitometer and the ImageJ application (rsbweb.nih.gov/ij/). Computational phylogenetic analyses were performed using Phylogeny software (http://phylogeny.lirmm.fr) [53]. Multiple protein sequence alignments were conducted with ClustalW program and phylogenetic trees were inferred by maximum likelihood statistical methods using a bootstrapping procedure of 1000 iterations.

### Phenotypic analysis

Vegetative sporulation, growth rate, carotene production and virulence measurements were carried out as previously described [14, 19, 36, 54]. Interactions between different strains of *M*. *circinelloides* and J774A.1 macrophage cells were carried out in L15 medium, during 4 hours at 37°C. Regarding polarity index, ten images were taken from each interaction and a total of fifty germinating spores were measured from each image with ImageJ [55]. From the same images, germination was calculated considering germinated spores all those that presented a protuberant bud from the spherical spore. For the phospholipase D activity measurements, spores of the wild type strain and mutant *mcplD* were grown in MMC medium at pH = 4.5 during six hours. The mycelia from five biological replicates were filtrated, washed and weighted to perform the assay with exactly 100 mg of biomass from each strain.

The virulence assays in *G*. *mellonella* were performed by injection of 5 μl of phosphate buffered saline (PBS) containing 2000 spores or 20,000 yeast cells into the wax moth larvae (10 per strain). For the murine host model, groups of 8 four-week-old OF1 male mice (Charles River, Criffa S.A., Barcelona, Spain) weighing 30 g were used. Mice were immunosuppressed 2 days prior to the infection by intraperitoneal (i.p.) administration of 200 mg/kg of body weight of cyclophosphamide and once every 5 days thereafter. Animals were housed under standard conditions with free access to food and water. Mice were challenged intravenously (i.v.) via the lateral tail vein with a suspension consisting on 1x10^5^ sporangiospores or 1x10^5^ yeast cells per animal. Animals were checked twice daily for 20 days. Surviving animals at the end of the experimental period or those meeting criteria for discomfort were euthanized by CO_2_ inhalation. Significance of mortality rate data was evaluated by using the Kaplan-Meier (Graph Pad Prism 4.0 for Windows; GraphPad Software, San Diego California USA). Differences were considered statistically significant at a *P* value of <0.05.

### Fungal burden quantification and genotyping

For absolute DNA quantification and genotyping, organs were ground up on liquid nitrogen and gDNA was extracted as previously described [56]. For DNA quantification by real-time PCR (qRT-PCR) specific primers of *M*. *circinelloides* chitin synthase gene (ID153118) and mice *β*2 microglobulin gene (ID12010) were used (S2 Table). Samples analyses were carried out in triplicate in 15 μl PCR reactions containing 180 ng of test sample gDNA form three individuals using SybrGreen kit (Fast SYBR Green Master Mix -ABI) in a StepOne Real-Time PCR System (ABI). gDNA from non-infected mice was used as negative control. Relative amount of fungal and mice gDNA was quantified on the basis of their standard curves, elaborated with known fungal DNA concentrations (0.005 ng—10 ng) in a background of 150 ng of non-infected mice gDNA and mice DNA concentrations (1 ng—200 ng) and their corresponding amplification cycle threshold (Ct).

### Ethics statement

Animal care procedures were supervised and approved by the Universitat Rovira i Virgili Animal Welfare and Ethics Committee. The experimental animal facilities are registered under reference T9900003 of the Generalitat de Catalunya in compliance with the regulations of Real Decreto 53/2013, of February 1st (BOE of 8 February). Procedures included into the project number 280 were supervised and approved by L. Loriente Sanz (ID 39671243) of the Veterinary and Animal Welfare Advisory of the Universitat Rovira i Virgili Animal Welfare and Ethics Committee (Reus, Spain).

## Supporting Information

S1 Fig Virulence assays of the twenty-six transformants showing abnormal phenotypes. HRG: highly reduced growth; SG: satellite growth; RG: reduced growth; LVS: low vegetative sporulation; YLG: yeast-like growth. Virulence assays of the YLG1 transformant compared to the wild type strains were performed using yeast cells in the infections (y). NRRL3631 and MU402+pMAT1700 were used as avirulent and virulent control strains, respectively.
(TIF)

S2 Fig Identity of the products encoded by the genes *mcmyo5 and mcplD*. (A) Phylogenetic study of fungal phospholipase genes and their relationship with *mcplD*. Names and ID number of these fungal phospholipases are listed in S4 Table. (B) Phylogenetic study of fungal myosin genes and their relationship with *mcmyo5*. Names and ID number of these myosins are listed in S3 Table.
(TIF)

S3 Fig Disruption of genes *mcclasp*, *mcmyo5 and mcplD*. (A) Schematic representation of wild-type (WT) and mutant (MUT) loci after homologous recombination with the disruption fragments of genes *mcclasp* (left), *mcmyo5* (middle) and *mcplD* (right). The position of the probes used (a, b and c) and the expected sizes of the restriction fragments are indicated; *pyrG* selectable marker; dashed lines, sequences not included in the disruption fragment. (B) Southern blot analysis of the wild-type strain R7B and transformants obtained with the disruption fragments after ten vegetative cycles in selective medium. Genomic DNA (1 μg) was digested with *SalI* (left, gene *mcclasp*), *BglII* (middle, gene *mcmyo5*) and *PuvII* (right, gene *mcplD*) and hybridized with probes a, b and c, which recognized wild-type and disrupted alleles but could discriminate between them. The positions and sizes of the GeneRuler DNA ladder mixture (M) (Fermentas) size markers are indicated.
(TIF)

S4 Fig Phospholipase D activity assay in mutant *ΔmcplD*. The total phospholipase D activities in wild type (R7B) and mutant *ΔmcplD* were measured using a commercial Phospholipase D Assay Kit (Sigma-Aldrich). In this assay, PLD hydrolyzes phosphatidylcholine to choline, which is determined using choline oxidase resulting in a colorimetric (570nm) product, proportional to the PLD activity in the sample.
(TIF)

S5 Fig Virulence tests of the *ΔmcplD* and *Δmcmyo5*^*(-)(+)*^ mutants of *M*. *circinelloides* in a murine host model. (A) Virulence assays using spores of wild type strains and the mutant *ΔmcplD*. Mice were injected with 1x10^5^ sporangiospores. (B) Virulence assays using yeast cells of wild type strains and the mutant *Δmcmyo5*^*(-)(+)*^. Injections contained 1x10^5^ yeast cells.
(TIF)

S6 Fig Segregation of the heterokaryon *Δmcmyo5*^*(-)(+)*^ under no selective conditions. (A) segregation of the heterokaryon *Δmcmyo5*^*(-)(+)*^ in MMC medium. A colony of the heterokaryon *Δmcmyo5*^*(-)(+)*^ was grown either with (right) or without uridine (left) in MMC medium during 3 days. Under no selective conditions (with uridine), the heterokaryon *Δmcmyo5*^*(-)(+)*^ segregates and produces patches reverting to the wild type phenotype. (B) Segregation of the heterokaryon *Δmcmyo5*^*(-)(+)*^ in retrieved CFUs from infected mice. Two types of retrieved CFUs, from agonizing mice (aCFUs) or apparently healthy mice (hCFUs) were analyzed in a southern blot similar to the assay described in S2 Fig. (C) Densitometric analysis of the bands corresponding to the mutated nuclei (blue arrow in B) and wild type nuclei (red arrow in B).
(TIF)

S7 Fig Size of the infecting inoculum. (A) Spore sizes of the *mcclasp* and *mcplD* mutants. (B) Yeast cell sizes of the *mcmyo5* mutant. Yeast cells were obtained after growing mycelia in liquid MMC pH 4.5 under anaerobiosis conditions during 24h.
(TIF)

S1 Table RNAi induced by high-throughput silencing plasmid pMAT1700 in *M*. *circinelloides*. Plasmid pMAT1700 was generated by cloning a synthetic insert between *SacI/KpnI* restriction sites of pBluescript SK+ (Promega). This insert contains two inverted *M*. *circinelloides* strong promoters, P*zrt1* (1 kb) and P*gpd1* (0.76 kb), a MCS and a 0.5 kb fragment of the 5’ end of *carB* gene.
(DOCX)

S2 Table Oligonucleotides used in this work. Red letters represent added restriction enzyme sites to facilitate cloning of the PCR products.
(DOCX)

S3 Table List of names and ID numbers of the fungal myosins studied in S2 Fig. (DOCX)

S4 Table List of names and ID numbers of the fungal phospholipases studied in S2 Fig. (DOCX)
