# Supplementary figures and images for: RNAi-Based Functional Genomics Identifies New Virulence Determinants in Mucormycosis
Source: PLoS Pathog. 2017 Jan 20;13(1):e1006150. doi: 10.1371/journal.ppat.1006150 (PMC5287474; doi:10.1371/journal.ppat.1006150)

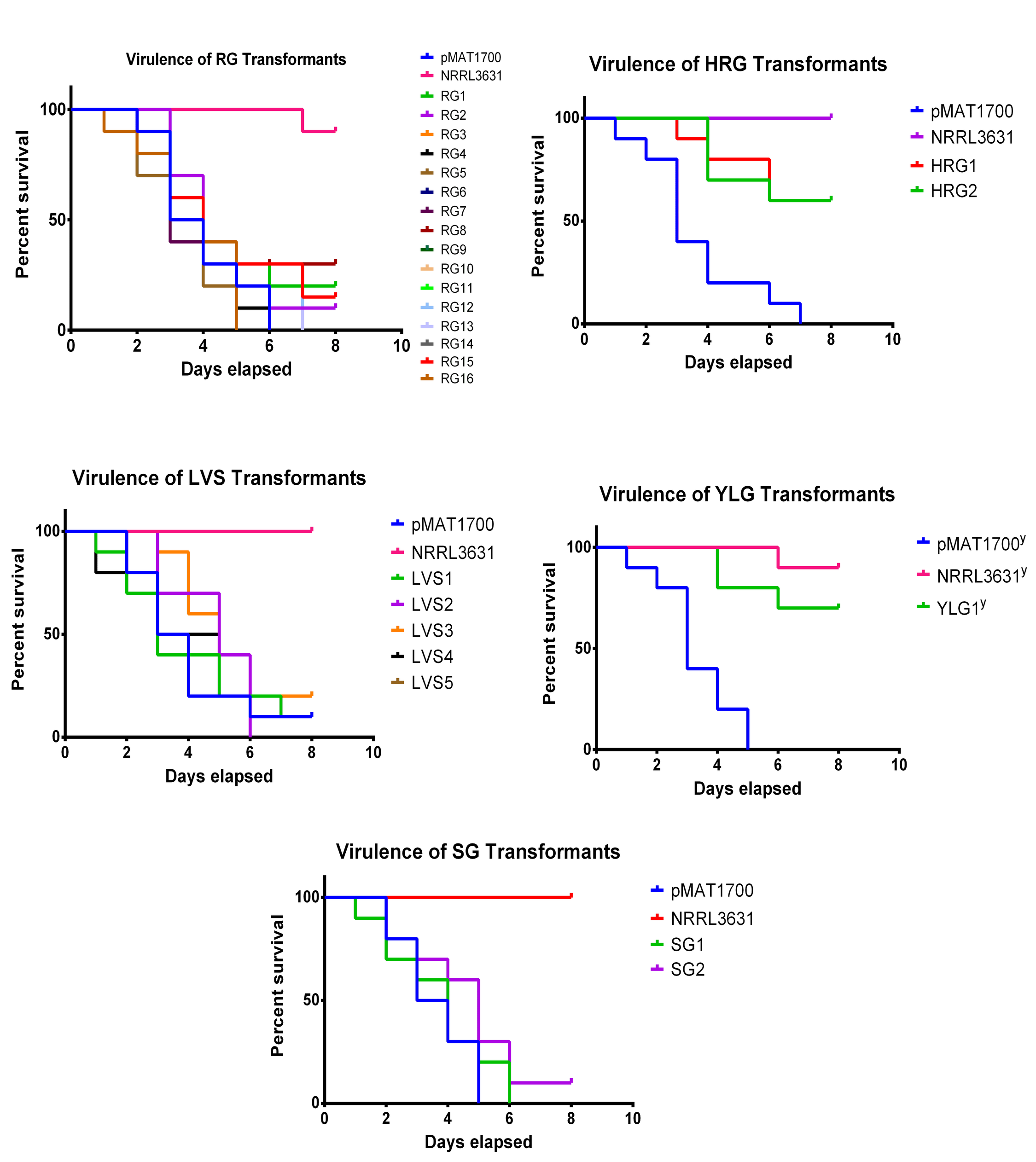

Supplement: S1 Fig — HRG: highly reduced growth; SG: satellite growth; RG: reduced growth; LVS: low vegetative sporulation; YLG: yeast-like growth. Virulence assays of the YLG1 transformant compared to the wild type strains were performed using yeast cells in the infections (y). NRRL3631 and MU402+pMAT1700 were used as avirulent and virulent control strains, respectively. (TIF) [file ppat.1006150.s001.tif]

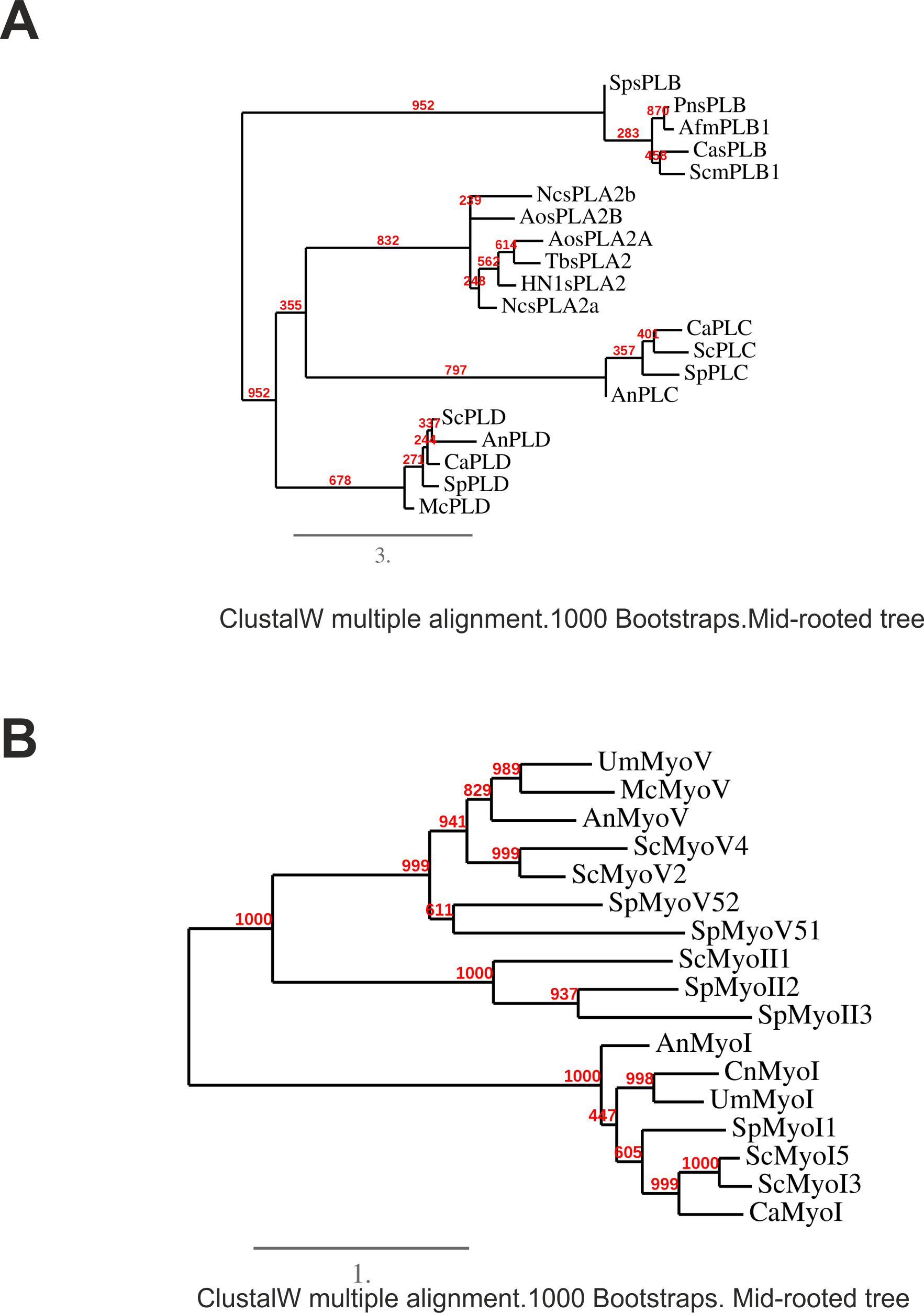

Supplement: S2 Fig — (A) Phylogenetic study of fungal phospholipase genes and their relationship with mcplD. Names and ID number of these fungal phospholipases are listed in S4 Table. (B) Phylogenetic study of fungal myosin genes and their relationship with mcmyo5. Names and ID number of these myosins are listed in S3 Table. (TIF) [file ppat.1006150.s002.tif]

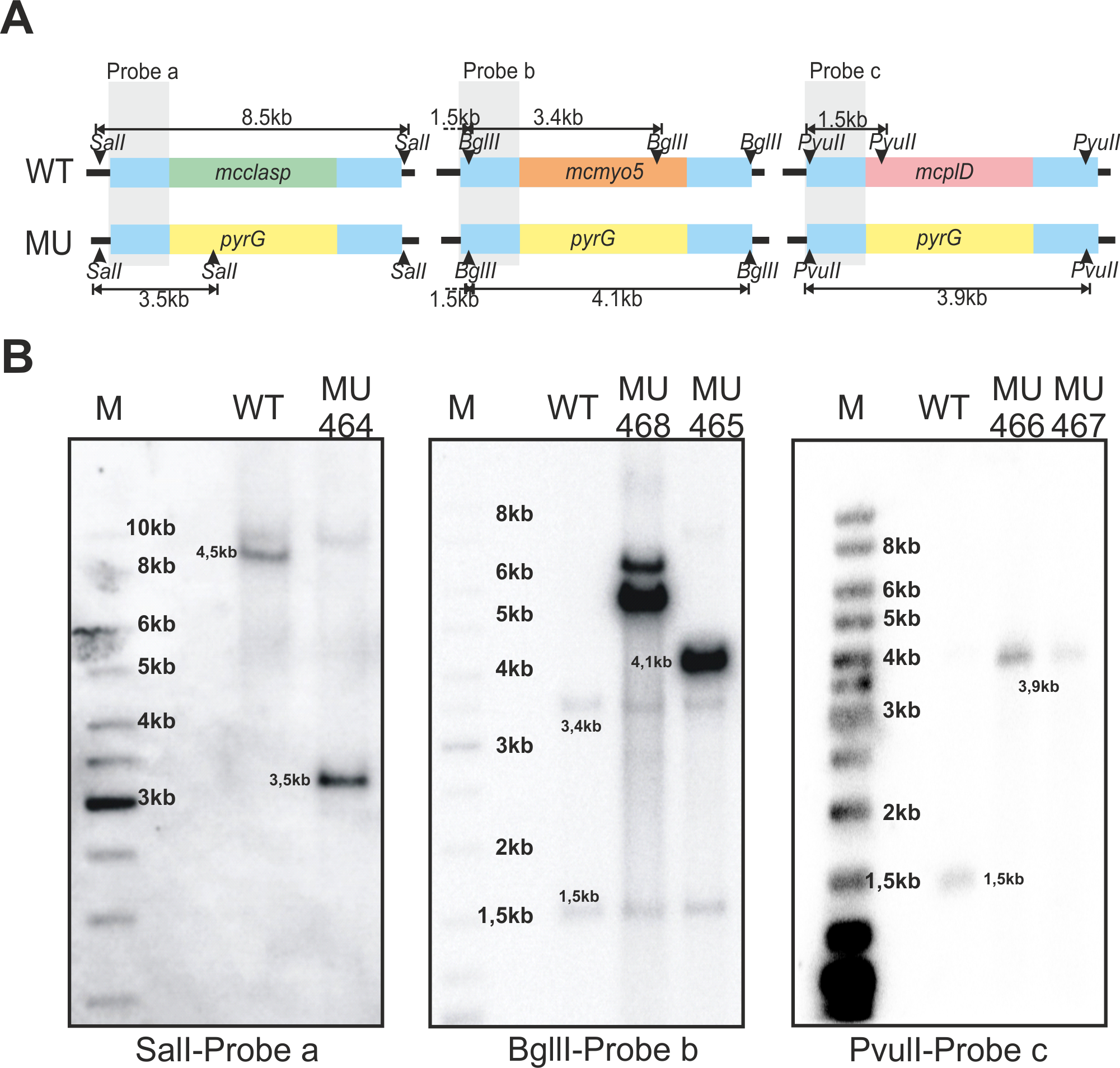

Supplement: S3 Fig — (A) Schematic representation of wild-type (WT) and mutant (MUT) loci after homologous recombination with the disruption fragments of genes mcclasp (left), mcmyo5 (middle) and mcplD (right). The position of the probes used (a, b and c) and the expected sizes of the restriction fragments are indicated; pyrG selectable marker; dashed lines, sequences not included in the disruption fragment. (B) Southern blot analysis of the wild-type strain R7B and transformants obtained with the disruption fragments after ten vegetative cycles in selective medium. Genomic DNA (1 μg) was digested with SalI (left, gene mcclasp), BglII (middle, gene mcmyo5) and PuvII (right, gene mcplD) and hybridized with probes a, b and c, which recognized wild-type and disrupted alleles but could discriminate between them. The positions and sizes of the GeneRuler DNA ladder mixture (M) (Fermentas) size markers are indicated. (TIF) [file ppat.1006150.s003.tif]

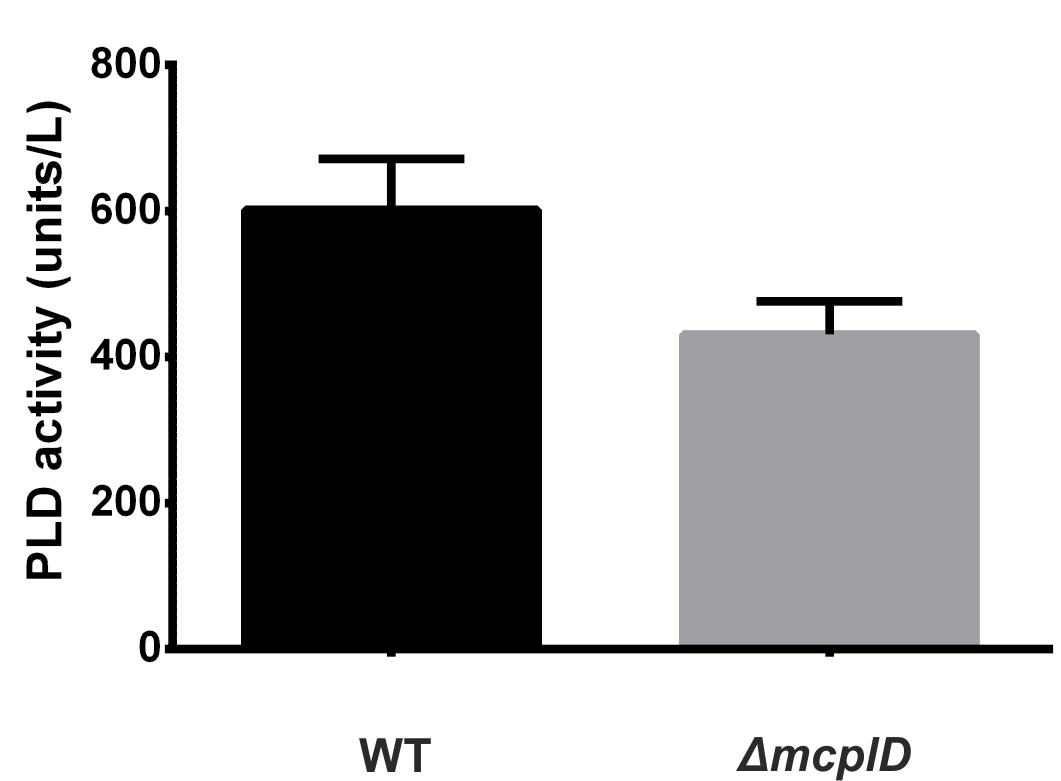

Supplement: S4 Fig — The total phospholipase D activities in wild type (R7B) and mutant ΔmcplD were measured using a commercial Phospholipase D Assay Kit (Sigma-Aldrich). In this assay, PLD hydrolyzes phosphatidylcholine to choline, which is determined using choline oxidase resulting in a colorimetric (570nm) product, proportional to the PLD activity in the sample. (TIF) [file ppat.1006150.s004.tif]

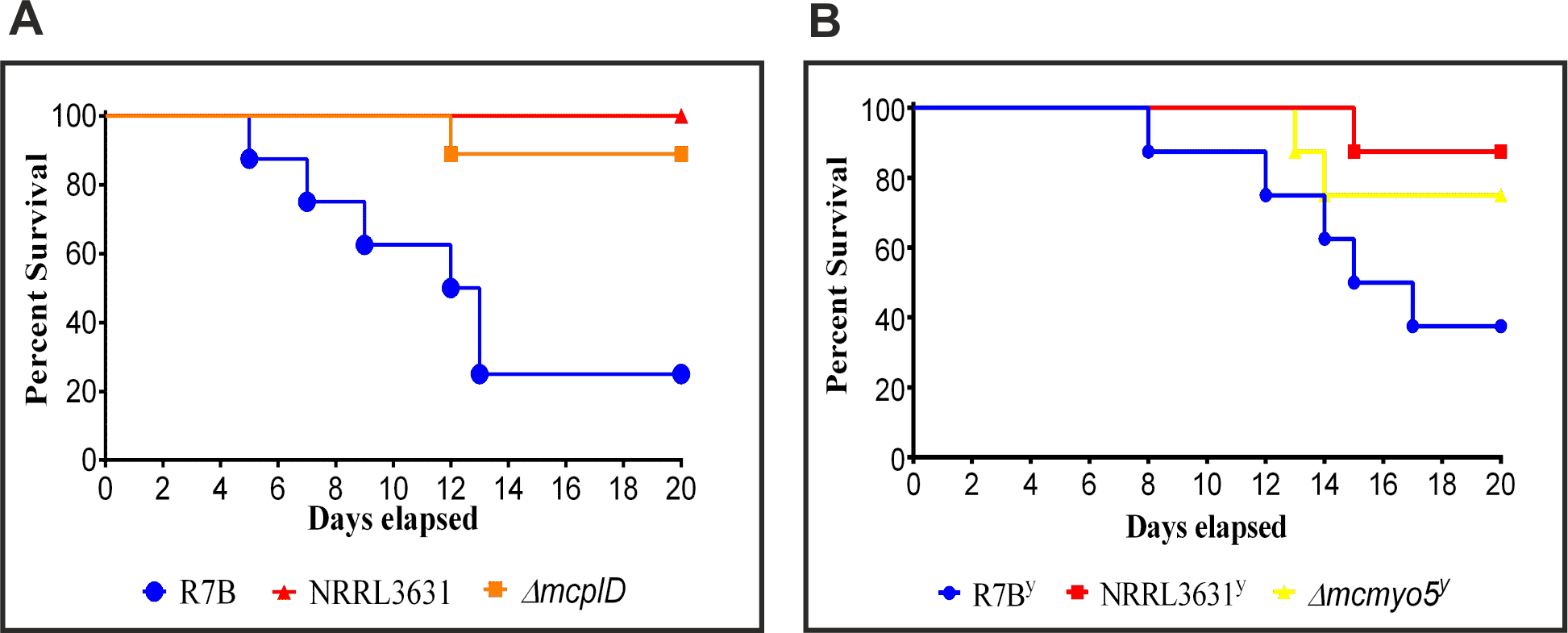

Supplement: S5 Fig — (A) Virulence assays using spores of wild type strains and the mutant ΔmcplD. Mice were injected with 1x10^5^ sporangiospores. (B) Virulence assays using yeast cells of wild type strains and the mutant Δmcmyo5(-)(+). Injections contained 1x10^5^ yeast cells. (TIF) [file ppat.1006150.s005.tif]

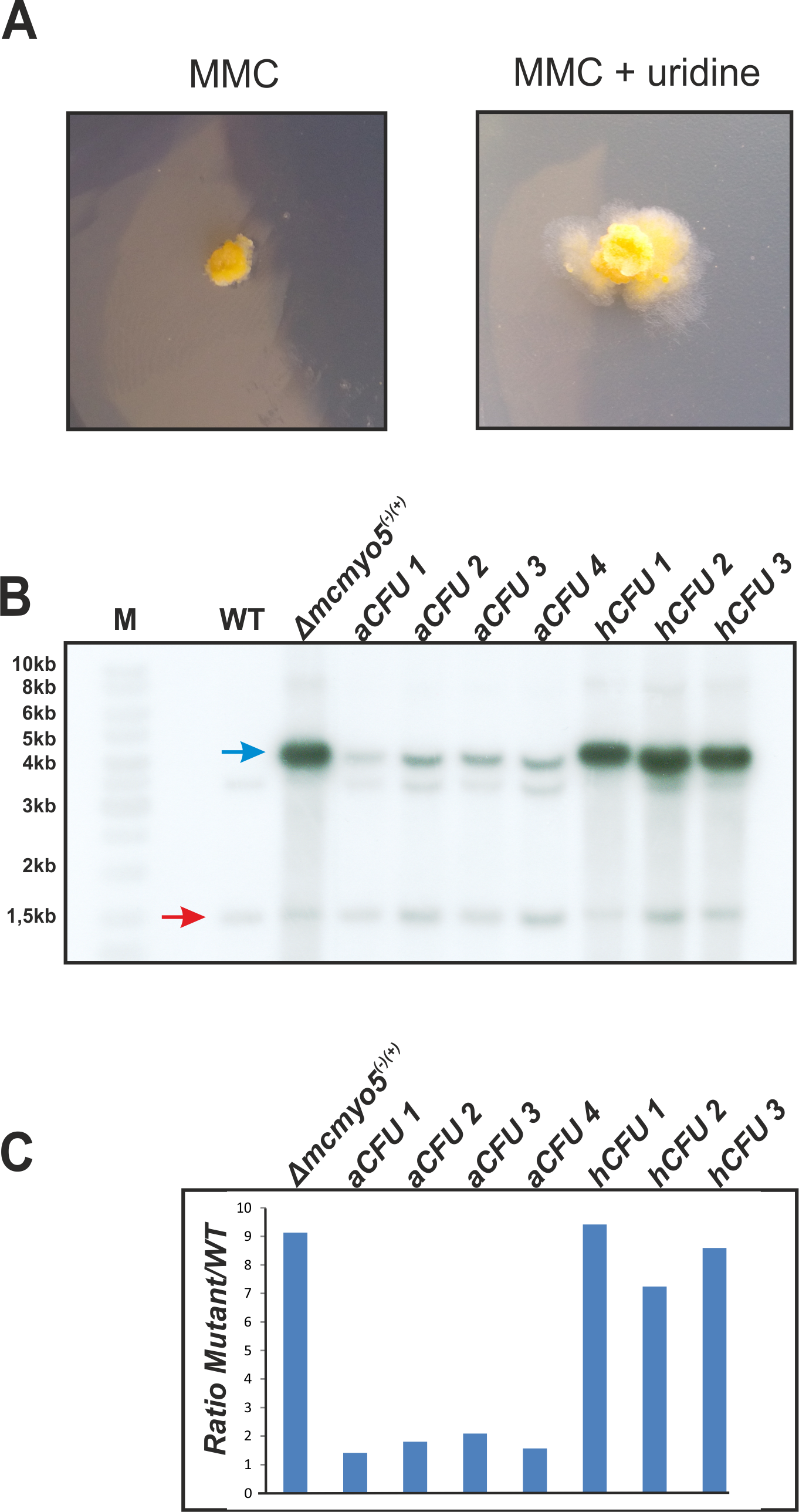

Supplement: S6 Fig — (A) segregation of the heterokaryon Δmcmyo5(-)(+) in MMC medium. A colony of the heterokaryon Δmcmyo5(-)(+) was grown either with (right) or without uridine (left) in MMC medium during 3 days. Under no selective conditions (with uridine), the heterokaryon Δmcmyo5(-)(+) segregates and produces patches reverting to the wild type phenotype. (B) Segregation of the heterokaryon Δmcmyo5(-)(+) in retrieved CFUs from infected mice. Two types of retrieved CFUs, from agonizing mice (aCFUs) or apparently healthy mice (hCFUs) were analyzed in a southern blot similar to the assay described in S2 Fig. (C) Densitometric analysis of the bands corresponding to the mutated nuclei (blue arrow in B) and wild type nuclei (red arrow in B). (TIF) [file ppat.1006150.s006.tif]

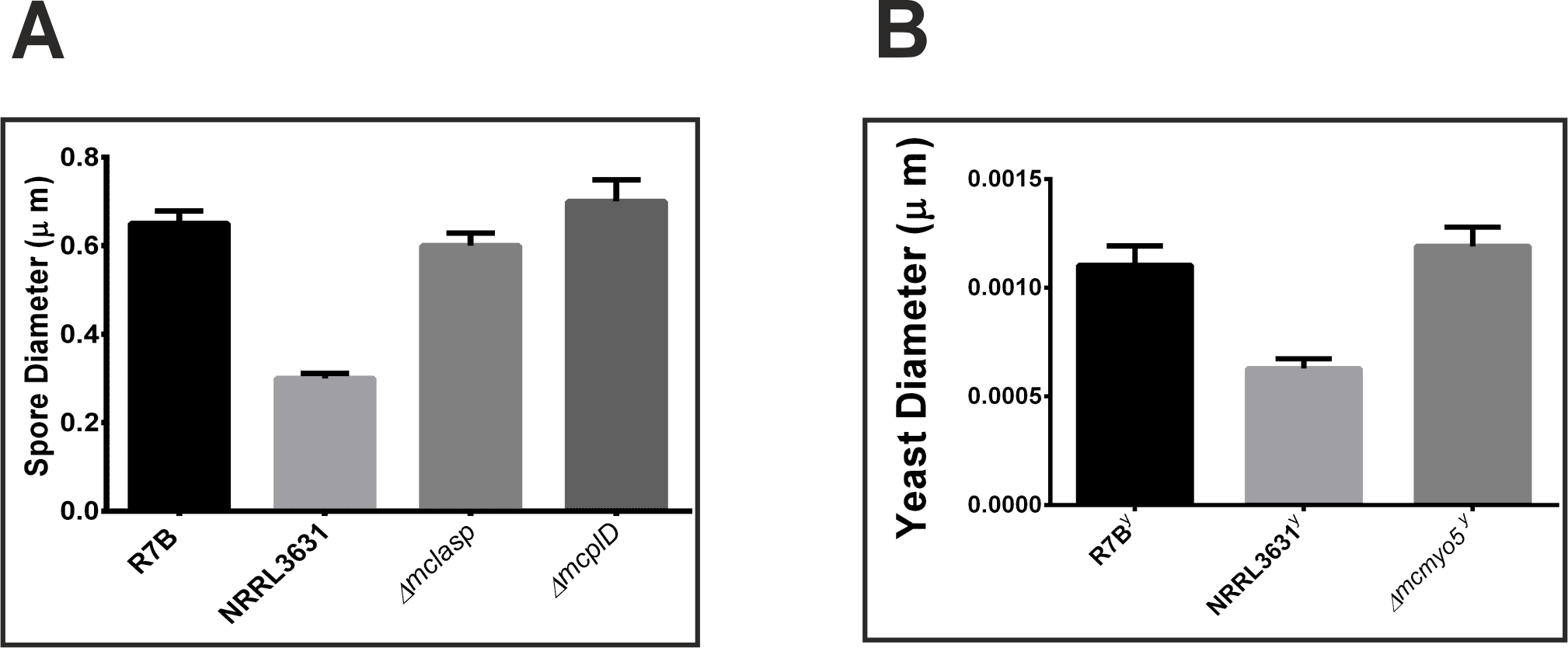

Supplement: S7 Fig — (A) Spore sizes of the mcclasp and mcplD mutants. (B) Yeast cell sizes of the mcmyo5 mutant. Yeast cells were obtained after growing mycelia in liquid MMC pH 4.5 under anaerobiosis conditions during 24h. (TIF) [file ppat.1006150.s007.tif]
