# Supplementary material for: RNAi-Based Functional Genomics Identifies New Virulence Determinants in Mucormycosis
Source: PLoS Pathog. 2017 Jan 20;13(1):e1006150. doi: 10.1371/journal.ppat.1006150 (PMC5287474; doi:10.1371/journal.ppat.1006150)
Supplement: S1 Table — Plasmid pMAT1700 was generated by cloning a synthetic insert between SacI/KpnI restriction sites of pBluescript SK+ (Promega). This insert contains two inverted M. circinelloides strong promoters, Pzrt1 (1 kb) and Pgpd1 (0.76 kb), a MCS and a 0.5 kb fragment of the 5’ end of carB gene. (DOCX) [file ppat.1006150.s008.docx]

| **Plasmids** |  | **Nº of transformants** |  | **Silencing**  **frequencies (%)** |
| --- | --- | --- | --- | --- |
|  | **Albino** | **Bright yellow** | **Total** |  |
| **pMAT1700(library)** | **45096** | **6560** | **51657** | **87%** |
| **pMAT1700(empty)** | **2266** | **2979** | **5245** | **43%** |
| **pMAT1701(*carB-*)** | **0** | **4985** | **4985** | **0%** |

**S1 Table**
