# Supplementary material for: RNAi-Based Functional Genomics Identifies New Virulence Determinants in Mucormycosis
Source: PLoS Pathog. 2017 Jan 20;13(1):e1006150. doi: 10.1371/journal.ppat.1006150 (PMC5287474; doi:10.1371/journal.ppat.1006150)
Supplement: S2 Table — Red letters represent added restriction enzyme sites to facilitate cloning of the PCR products. (DOCX) [file ppat.1006150.s009.docx]

| **NAME** | **SEQUENCE** | **USE** |
| --- | --- | --- |
| **peuka-1** | CATGAAGTGTGAGACATTGCG | Target sequencing |
| **carbsal-1** | TTCGTCGACAGCGAACTCGGTGTACTGCAAC | Target sequencing |
| **FYL1** | TCGCGGCCGCACCACGGTGAAAGCGCTGTTGGAG | Plamid pMAT828 |
| **RYL1** | CTCTCGAGCTTCAGGAGTAGCACAAGCTGTTCAAG | Plamid pMAT828 |
| **FYL1U** | AAGCCGACGAGCCTGTGGCAGATG | *Mcmyo5* disruption |
| **RYL1D** | CTCACCATCCTCACATTCAACGAAACAAGG | *Mcmyo5* disruption |
| **FYL1N** | GTGCTTGGATTGAACCCGCTGCCCTT | *Mcmyo5* disruption |
| **RYL1N** | GGCATTGGAAGAGCAACTAGCTTTTGGAG | *Mcmyo5* disruption |
| **RYL1-pyrG** | CAAGTACCAATGCTGAGGCAGACCATCTTACACATCCAAGTGCCTGA | *Mcmyo5* disruption |
| **FYL1-pyrG** | CGATAGCATGGCCAGTGTACGACACCCAAGCCTCGTCTTCGTCTT | *Mcmyo5* disruption |
| **FYL1.2** | TCGCGGCCGCACCACGGTGAAAGCGCTGTTGGAG | Plasmid pMAT798 |
| **RYL1.2** | CTCTCGAGCTTCAGGAGTAGCACAAGCTGTTCAAG | Plasmid pMAT798 |
| **FYL10.1** | ATGCGGCCGCCAAAGATGAATCCACAGACGTAGATCCTGT | Plasmid pMAT823 |
| **RYL10.1** | ATCTCGAGGTGTTCCTGCTGCATCTGAGACGAG | Plasmid pMAT823 |
| **FYL10U** | TACCGACGGGCCATGCCTTTGAG | *mcclasp* disruption |
| **RYL10D** | GACGAGGATCTTTCTTCTGCCCAGC | *mcclasp* disruption |
| **FYL10** | ATCCCTTGCGCGAACCATTGTCTCACG | *mcclasp* disruption |
| **RYL10** | TTGATGGGGTGAAAGAGAGGTGGAGATG | *mcclasp* disruption |
| **RYL10-pyrG** | CAAGTACCAATGCTGAGGCAGTGTGTTGGTACCTGAAGGGTGCCT | *mcclasp* disruption |
| **FYL10-pyrG** | CGATAGCATGGCCAGTGTACGCCTTGGTCGCCTTTCACGAAGTTC | *mcclasp* disruption |
| **FYL10.2** | TTGCGGCCGCTCCAGCTATGAGCAAGACTTTACATCCATC | Plasmid pMAT824 |
| **RYL10.2** | TTCTCGAGCCATGCCTTTGAGCGTCTTAGATGCC | Plasmid pMAT824 |
| **FYL10.3** | ATGCGGCCGCCGTCATTAGCGTCCTTGATACCTTCTTCAG | Plasmid pMAT825 |
| **RYL10.3** | ATCTCGAGCACAGTGGAACTCAGTGATAAACACACTG | Plasmid pMAT825 |
| **PLDU** | AAAGCGAGGCGTGCTGAATCGGT | *mcplD* disruption |
| **FPLD** | CTCGAGATGATCAAGAAATGGGTAGGAAATTATAGACAGC | *mcplD* disruption |
| **RPLD** | GCGGCCGCCACAAAGGCTGCTTCATGTAGCTC | *mcplD* disruption |
| **FPLD-pyrG** | CGATAGCATGGCCAGTGTACCGTGGACTGATGTTTCTATGCGTCT | *mcplD* disruption |
| **RPLD-pyrG** | CAAGTACCAATGCTGAGGCAGCGAGATTGCTTTGGATCACGGTCT | *mcplD* disruption |
| **RPLD1** | CGAGATTGCTTTGGATCACGGTCT | *mcplD* disruption |
| **ChsV-1** | CAAGGACGAAAAGAGAGTAAC | *qPCRs, fungal burden* |
| **ChsV-3** | TGTTGGTAGTTGTGATAATCGT | *qPCRs, fungal burden* |
| **B2m-F** | TTTTCATCTGTCTTCCCCTGT | *qPCRs, fungal burden* |
| **B2m-R** | GTATGTATCAGTCTCAGTGGG | *qPCRs, fungal burden* |
