# Supplementary material for: RNAi-Based Functional Genomics Identifies New Virulence Determinants in Mucormycosis
Source: PLoS Pathog. 2017 Jan 20;13(1):e1006150. doi: 10.1371/journal.ppat.1006150 (PMC5287474; doi:10.1371/journal.ppat.1006150)
Supplement: S3 Table — (DOCX) [file ppat.1006150.s010.docx]

**S3 Table.**

| **Name** | **Ac. Nº UNIPROT** | **Gene** | **Organism** |
| --- | --- | --- | --- |
| McMyoV | A0A162QBL5 | *mcmyo5* | *M. circinelloides* |
| ScMyoV2 | P19524 | *MYO2* | *Saccharomyces cerevisiae* |
| ScMyoV4 | P32492 | *MYO4* | *S. cerevisiae* |
| SpMyoV51 | O74805 | *myo51* | *Schizosaccharomyces pombe* |
| SpMyoV52 | O94477 | *myo52* | *S. pombe* |
| AnMyoV | Q5AS68 | *myoE* | *Aspergillus nidulans* |
| UmMyoV | Q7Z8J7 | *myo5* | *Ustilago maydis* |
| ScMyoI3 | P36006 | *MYO3* | *S. cerevisiae* |
| ScMyoI5 | Q04439 | *MYO5* | *S. cerevisiae* |
| SpMyoI1 | Q9Y7Z8 | *myo1* | *S. pombe* |
| CaMyoI | Q59MQ0 | *MYO5* | *Candida albicans* |
| AnMyoI | Q00647 | *myoA* | *A. nidulans* |
| UmMyoI | Q7Z8J6 | *myo1* | *U. maydis* |
| CnMyoI | P0CP00 | *MYO1* | *Cryptococcus neoformans* |
| ScMyoII1 | P08964 | *MYO1* | *S. cerevisiae* |
| SpMyoII2 | Q9USI6 | *myo2* | *S. pombe* |
| SpMyoII3 | O14157 | *myo3* | *S. pombe* |
