# Supplementary material for: RNAi-Based Functional Genomics Identifies New Virulence Determinants in Mucormycosis
Source: PLoS Pathog. 2017 Jan 20;13(1):e1006150. doi: 10.1371/journal.ppat.1006150 (PMC5287474; doi:10.1371/journal.ppat.1006150)
Supplement: S4 Table — (DOCX) [file ppat.1006150.s011.docx]

**S4 Table.**

| **Name** | **Ac. Nº UNIPROT** | **Gene** | **Organism** |
| --- | --- | --- | --- |
| McPLD | A0A162ZS82 | *mcplD* | *M. circinelloides* |
| ScPLD | P36126 | *SPO14* | *S. cerevisiae* |
| SpPLD | Q09706 | *pld1* | *S. pombe* |
| CaPLD | O74136 | *caPLD* | *C. albicans* |
| AnPLD | Q5AYB8 | AN6712 | *A. nidulans* |
| ScPLC | P32383 | *PLC1* | *S. cerevisiae* |
| SpPLC | P40977 | *plc1* | *S. pombe* |
| CaPLC | O13433 | *PLC1* | *C. albicans* |
| AnPLC | Q5BFL6 | AN0664 | *A. nidulans* |
| ScmPLB1 | P39105 | *PLB1* | *S. cerevisiae* |
| SpsPLB | P78854 | *plb1* | *S. pombe* |
| CasPLB | Q9UWF6 | *PLB1* | *C. albicans* |
| AfmPLB1 | B0Y665 | *plb1* | *Aspergillus fumigatus* |
| PnsPLB | P39457 | *plb1* | *Penicillium notatum* |
| NcsPLA2a | Q1K6W0 | NCU06650 | *Neurospora crassa* |
